# Multi-reference global registration of individual A-lines in adaptive optics optical coherence tomography retinal images

**DOI:** 10.1117/1.JBO.26.1.016001

**Published:** 2021-01-06

**Authors:** Kazuhiro Kurokawa, James A. Crowell, Nhan Do, John J. Lee, Donald T. Miller

**Affiliations:** a Indiana University, School of Optometry, Bloomington, Indiana, United States; b Purdue School of Engineering and Technology, Indiana University-Purdue University Indianapolis, Indianapolis, Indiana, United States; c Google, Mountain View, California, United States

**Keywords:** three-dimensional registration, image registration, adaptive optics, optical coherence tomography, parallel processing

## Abstract

**Significance:** Adaptive optics optical coherence tomography (AO-OCT) technology enables non-invasive, high-resolution three-dimensional (3D) imaging of the retina and promises earlier detection of ocular disease. However, AO-OCT data are corrupted by eye-movement artifacts that must be removed in post-processing, a process rendered time-consuming by the immense quantity of data.

**Aim:** To efficiently remove eye-movement artifacts at the level of individual A-lines, including those present in any individual reference volume.

**Approach:** We developed a registration method that cascades (1) a 3D B-scan registration algorithm with (2) a global A-line registration algorithm for correcting torsional eye movements and image scaling and generating global motion-free coordinates. The first algorithm corrects 3D translational eye movements to a single reference volume, accelerated using parallel computing. The second algorithm combines outputs of multiple runs of the first algorithm using different reference volumes followed by an affine transformation, permitting registration of all images to a global coordinate system at the level of individual A-lines.

**Results:** The 3D B-scan algorithm estimates and corrects 3D translational motions with high registration accuracy and robustness, even for volumes containing microsaccades. Averaging registered volumes improves our image quality metrics up to 22 dB. Implementation in CUDA™ on a graphics processing unit registers a 512×512×512 volume in only 10.6 s, 150 times faster than MATLAB™ on a central processing unit. The global A-line algorithm minimizes image distortion, improves regularity of the cone photoreceptor mosaic, and supports enhanced visualization of low-contrast retinal cellular features. Averaging registered volumes improves our image quality up to 9.4 dB. It also permits extending the imaging field of view (∼2.1×) and depth of focus (∼5.6×) beyond what is attainable with single-reference registration.

**Conclusions:** We can efficiently correct eye motion in all 3D at the level of individual A-lines using a global coordinate system.

## 1 Introduction

Adaptive optics-optical coherence tomography (AO-OCT) is a non-invasive, high-resolution method for obtaining three-dimensional (3D) volumetric images of the retina.^1^^–^^4^ These qualities may support earlier detection and improved monitoring of prevalent blinding retinal diseases, such as glaucoma, diabetic retinopathy, and age-related macular degeneration.^1^^–^^4^ However, analysis of AO-OCT data is impeded by two factors: First, the time to acquire a single volume is sufficient for it to be distorted by eye motions such as tremors, drifts, and microsaccades,^5^ creating artifacts that are many times larger than the cells being imaged.^6^ These motion artifacts must be measured and compensated for to permit proper analysis of the retinal tissue images. We address this problem by proposing a cascade of two registration algorithms that we call 3D B-scan registration^7^ and global A-line registration. Second, high-resolution AO-OCT systems generate terabytes of image data from a single imaging session, resulting in immense data sets and making motion artifact removal a lengthy process. We decrease processing time by implementing the 3D B-scan registration algorithm with parallel processing on a general-purpose graphics processing unit (GPGPU).^7^ Together, the two registration algorithms allow us to compensate efficiently and accurately for both lateral and axial eye motions. The algorithms correct for XYZ translational eye movements, torsional eye movements, and variations in image magnification, and generate global motion-free coordinates.

Numerous algorithms have been developed for registering images from adaptive optics scanning laser ophthalmoscopes (AO-SLO)^8^^–^^11^ and AO-OCT.^12^^–^^14^ These are based universally on strip-wise registration of two-dimensional (2D) en face images. Strip-wise registration specifically corrects motion artifacts that appear in the en face image, which is the most common view of the retina obtained with AO ophthalmoscopes. This approach typically requires that images are decomposed into strips that are sufficiently narrow and acquired sufficiently quickly that they are individually free of motion artifacts (aside from an occasional microsaccade). Motion artifacts are therefore assumed to consist of rigid displacements of these strips that can vary in magnitude and direction. These displacements can be measured and corrected in a target volume by cross-correlating each strip from the en face image of that volume to multiple strips from the en face image of a reference volume.

When applied to volumes, strip-wise approaches are computationally inherently efficient in that the entire volume is registered by processing only a small fraction of the volume data, i.e., a 2D en face projection, typically through the photoreceptor layer. This layer has been historically used for registration as it contains the brightest reflections and hence the strongest signals. However, computational efficiency comes at the expense of discarding most of the information in the volume, which could be used for improving the registration. For our 3D B-scan registration algorithm, we take advantage of this additional information by extending the cross-correlation regions into the third dimension (depth), correlating B-scans instead of en face strips. Making use of the additional information outside the photoreceptor layer confers two important advantages. First, it permits us to trade an increased extension in-depth for a decrease in width. Specifically, instead of single-pixel-deep strips we use fast B-scans as single-pixel wide slices extending the full depth of the volume. This allows us to better detect and correct rapidly varying motion artifacts. Second, the depth of focus of AO ophthalmoscopes is typically a small fraction of the retinal thickness. When we image cells outside the photoreceptor layer, we focus the system on those cells, decreasing the sharpness of en face images of the photoreceptors. Cross correlation of B-scans is inherently less sensitive to changes in focus as all cells contribute to the registration and those cells in focus have increased signal and sharpness.

Effective strip-wise and B-scan registration require the reference volume to contain minimal motion artifacts and maximal overlap with the image set to be registered. Considering that no volume is ever completely free of motion artifacts, it follows that volumes registered by this method are still distorted. While several approaches have been proposed to mitigate such residual distortion, e.g., incorporating an eye-tracking system^6^^,^^15^^–^^17^ and modifying the scan pattern,^18^^,^^19^ these may require additional hardware and add complexity and cost. Stevenson and Roorda^8^ and others^9^^,^^10^^,^^14^ presented a clever method for constructing a synthetic global reference from the complete set of motion traces for each strip within a set of registered images. This permits registration of all images to a global reference and can be done entirely in post-processing. This method is successful, though is most effective within the plane containing the 2D registration strips (e.g., photoreceptor layer) and does not compensate for both scaling and torsional errors even within that plane. The method is also limited to a single reference.

**Fig. 1:**
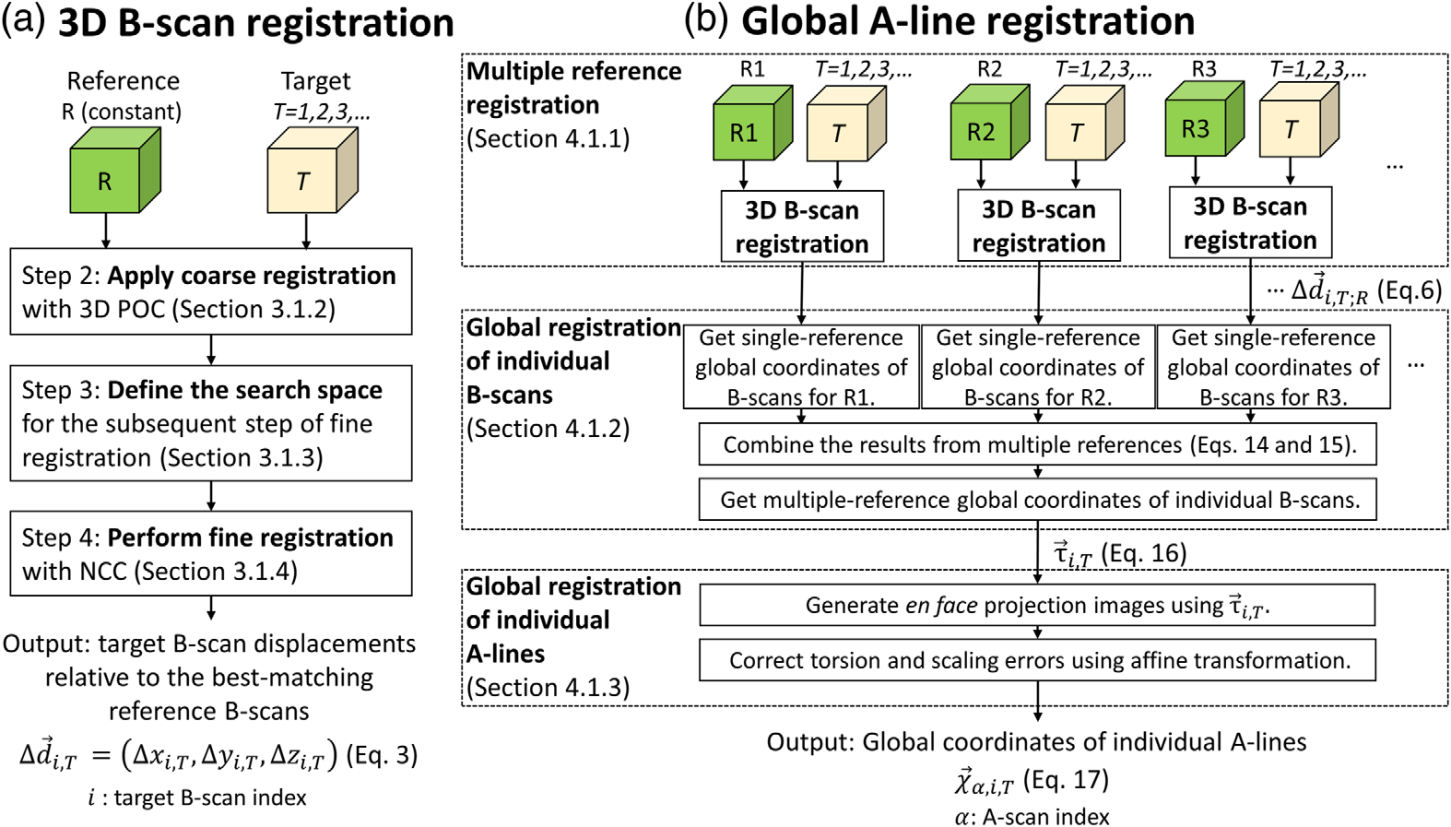
Flowchart of our registration method that cascades (a) a 3D B-scan registration algorithm with (b) a global A-line registration algorithm. The 3D B-scan registration algorithm registers multiple target volumes T=(1,2,…) to a single reference volume R. A coarse-to-fine registration approach improves processing speed and accuracy (Sec. 3.1.2.3), accelerated by parallel computing (Sec. A in Supplementary Material). Global A-line registration registers individual A-lines in all volumes to a global coordinate system. The approach consists of three steps: (1) multiple-reference registration to separately register all target volumes to each of multiple reference volumes (Sec. 4.1.1), (2) global registration of individual B-scans (Sec. 4.1.2) to derive a single mapping of individual B-scans to the multiple-reference global coordinate system, and (3) global registration of individual A-lines (Sec. 4.1.3) to correct en face affine transformations among volumes and estimate 3D global coordinates for individual A-lines. Abbreviations: POC, phase-only correlation; NCC, normalized cross-correlation.

We develop a more rigorous approach to addressing this global registration problem. We use our 3D B-scan registration algorithm to register target volumes to a reference volume. We test this algorithm by examining its effect on a number of image quality metrics. We then repeat the 3D B-scan registration by selecting multiple reference volumes to increase the number of registerable volumes. We use these registration results to derive a single global coordinate system that all individual B-scans from all volumes are mapped into. Finally, we correct for scaling and torsional errors to estimate 3D global coordinates for individual A-lines. We name this approach our global A-line registration algorithm. We test the global A-line algorithm by applying a slightly different set of image quality metrics to averaged volumes registered to the global coordinate system; this second set of metrics are intended to also measure enhancements in the regularity of the resulting images. Figure 1 shows a detailed flowchart of our registration method with steps referenced by section number in the paper.

Understanding our method and contrasting it against registration methods already in the literature requires an understanding of AO-OCT image acquisition and the impact eye motion has on it. These topics are introduced in Sec. 2. Section 3 presents our 3D B-scan registration algorithm and CUDA™ GPGPU implementation, which allows it to run ∼150 times faster than it would in MATLAB™ on a central processing unit (CPU). We experimentally evaluate our implementation by running it against a dataset of more than 300 volume images and measuring registration accuracy and run time both quantitatively and qualitatively. Section 4 presents our global A-line registration algorithm, which uses the output of the 3D B-scan registration algorithm. We derive the method from theory and experimentally evaluate it by running it against a dataset of more than 900 volume images and measuring the registration accuracy qualitatively and quantitatively. We use the combined registration method (3D B-scan registration plus global A-line registration) to obtain pristine images of retinal cellular structure, to increase the field of view (∼2×) over that of a single acquired AO-OCT volume, and to create through-focus AO-OCT volume images with preserved image sharpness over the full retinal thickness. The latter is made possible by increasing the effective depth of focus (∼5×) over that of a single acquired AO-OCT volume. In Sec. 5, we summarize the performance of our registration method, compare it to registration methods in the literature, and discuss its limitations.

Finally, this study focuses on the development rather than the use of a new registration method. Because of this, we found it most efficient to illustrate and validate our method using just two large datasets acquired on two subjects who had typical fixational ability. We have numerous other datasets that could have been used, and in fact, we now routinely use this method in all our imaging studies. To date, we have successfully registered images acquired on more than 40 subjects of different age, gender, and eye size, with different retinal diseases, and at numerous retinal eccentricities and time points. Some of these results can be found elsewhere.^4^^,^^20^^–^^25^ Also, while we limit the method to AO-OCT images, the method should be applicable to other types of 3D images, most obviously to OCT images (without AO), which are universally used in eye care practice.

## 2 AO-OCT Image Acquisition and Impact of Eye and Head Motion

Each AO-OCT image volume contains a unique pattern of lateral (x,y) and axial (z) distortions and is non-rigidly displaced in all 3D relative to other volumes because of random eye motion during fixation^5^ and subtle movements of the head. The nature of these distortions in each dimension is determined by an interaction between the temporal properties of the component of eye movement and the component of the OCT scan pattern in that dimension. The pixels within a single A-line of the volume (typically oriented in retinal depth, z) are captured simultaneously. A fast B-scan (x,z) consists of a set of A-lines spaced contiguously along the x axis; in our system, they are typically collected over a period of 0.3 to 1 ms. A complete volume (x,y,z) consists of a set of fast B-scans (x,z) displaced along the y axis and collected over 98 to 414 ms. A slow B-scan (y,z) is constructed from A-lines at the same x position along the y axis, whereas a C-scan (x,y) or en face image is constructed from rows of fast B-scans at the same depth and displaced along the y axis.

**Fig. 2:**
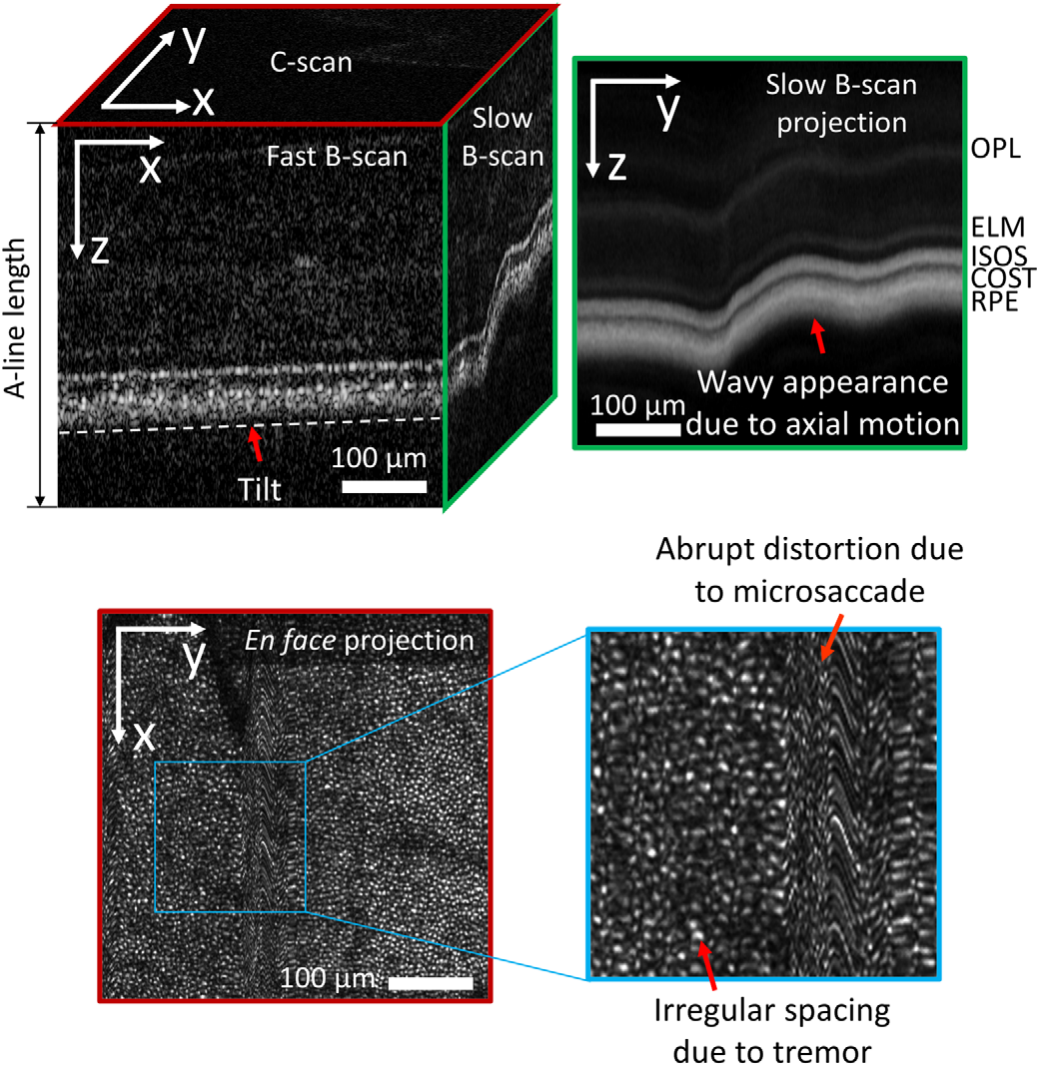
Eye motion artifacts in AO-OCT volume. (top left) A volume consists of a set number of fast B-scans spaced along the y axis with each fast B-scan consisting of a set number of A-lines spaced along the x axis. (top right) The slow B-scan projection image is the projection of the volume onto the yz plane by averaging all pixels along the x axis. The wavy looking pattern is due to axial motion. (bottom) Similarly, the en face projection image is the projection of the volume onto the xy plane by averaging all pixels along the z axis. The abrupt distortion is due to a microsaccade. The irregular spacing is due to tremor motion.

When we view a 2D slice through a volume, the appearance of eye motion artifacts depends on which slice is viewed: fast B-scan (x,z), slow B-scan (y,z), or C-scan (x,y). For example, the most commonly seen artifacts in fast B-scan images (x,z) is a tilt induced by a linear variation in the time-of-flight difference of the retinal reflections across the B-scan, as shown in the fast B-scan of Fig. 2 (top left). The time-of-flight variation is caused by displacement of the eye’s optical axis in its pupil plane relative to the imaging beam axis. However, in a slow B-scan image (y,z), head movement along the z axis is the dominant effect, causing slow (mean wave period ∼1  s) shifts or ripples of the image along the z axis, as shown by the slow B-scan and slow B-scan projection of Fig. 2 (top). Microsaccades are more evident in the en face image (x,y) and cause the largest and most abrupt shifts; they can traverse between several dozens and hundreds of photoreceptors over ∼25  ms,^5^ as shown in the en face projection in Fig. 2 (bottom). Tremors in the x and y dimensions cause small oscillatory shifts (∼2 to 3  μm in amplitude) with a duration of ∼11  ms,^5^ resulting in the distorted appearance of cone photoreceptors shown in Fig. 2 (bottom). While it is not evident in Fig. 2, drift movements cause moderate shifts—traversing roughly a dozen photoreceptors—over longer intervals (∼1  s).^5^

The process of image registration is also complicated by changes in head position that can give rise to scaling and rotation (torsion) of images. Measurable torsion across look-ins—caused by combination of changes in head position and compensatory torsional eye movements—is common,^26^^,^^27^ even in subjects experienced in the use of a bite bar. Also, the torsion error drifts over time (standard deviation of torsion error is ∼0.15  deg within a 4-s session, but an order of magnitude larger across sessions).^28^ Image scaling is primarily due to slight changes in position of the bite bar mount between imaging sessions; a slight change in position between two sessions (a year apart) of a longitudinal study^25^ gave rise to noticeable image scaling (the change in magnification is 2.2%/mm from a pivot conjugate plane). The effects of these motions are considerably more subtle and will be discussed in Sec. 3.

## 3 3D B-Scan Registration

Section 3.1 describes our 3D B-scan registration algorithm that uses a coarse-to-fine registration approach with a combination of phase correlation and normalized cross-correlation (NCC) to improve processing speed and accuracy.^7^ Section A in Supplementary Material summarizes our GPU-based acceleration techniques. Section 3.2 describes the experimental procedure used to evaluate the performance of our approach, and the experimental results.

### 3.1 Algorithm

**Fig. 3:**
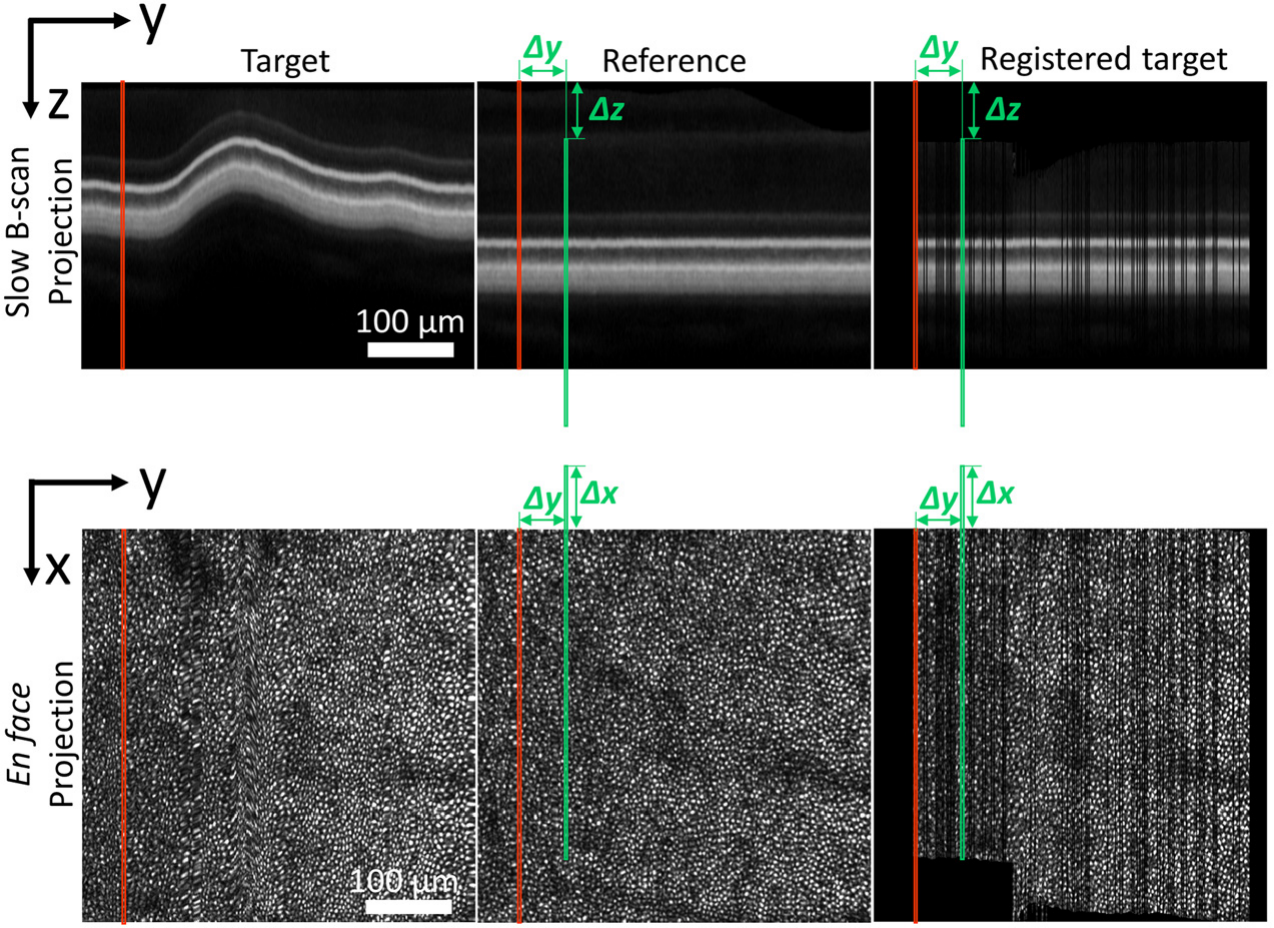
Example of 3D registering a single fast B-scan of an AO-OCT retinal volume to the best-matching fast B-scan in a reference volume. This figure shows the slow B-scan projection (top row) and en face projection (bottom row) images of the target volume (left column), reference volume (middle column), and registered target volume (right column). The red line in each image indicates the location in the target image of the fast B-scan to be registered. The green line indicates the location of the best-matching fast B-scan in the reference volume found by our 3D B-scan registration algorithm, displaced from the target location by (Δx,Δy,Δz). Target B-scans are shifted by this amount in the right column, registering the B-scans to the reference volume but creating gaps.

We assume that fast B-scans are rigid, i.e., that they were acquired in a short enough time (0.3 to 1 ms) to contain only negligible motion artifacts. Thus, for each fast B-scan in a target volume we can determine its offset (Δx,Δy,Δz) with respect to the best matching fast B-scan in a reference volume by 3D cross-correlation of individual fast B-scans between reference and target volumes. Figure 3 shows the concept of this 3D B-scan registration method.

We used a coarse-to-fine B-scan search to accelerate image registration. This strategy uses a faster but less accurate method to compute a coarse displacement estimate. This coarse estimate is then used to define a small search space for a subsequent fine estimate using a slower but more accurate technique. The overall 3D B-scan registration approach consists of five steps [Fig. 1(a)]:

1. Prepare AO-OCT volumes for 3D registration by filtering out noise, cropping volumes, and choosing a reference volume (Sec. 3.1.1).
2. Apply high-speed coarse registration with 3D phase-only correlation (POC)^29^ to obtain an approximate prediction of target B-scan image displacement relative to the best-matching B-scan in the reference volume (Sec. 3.1.2).
3. Define the search space for the subsequent step of fine registration by upsampling the coarsely-predicted B-scan displacements from the preceding step using a linear interpolator (Sec. 3.1.3).
4. Perform fine registration with NCC^30^ for accurate prediction of individual fast B-scan locations within the reduced search area (Sec. 3.1.4).
5. Iterate (2)–(4) to register other target volumes to the same reference volume and then filter the registration results and visualize the registered averaged high-quality AO-OCT volume (Sec. 3.1.5).

This correlation-based algorithm is well-suited to parallel processing, enabling all computations to be implemented in CUDA (Sec. A in Supplementary Material).

#### 3.1.1 Data preparation

AO-OCT volumes require six pre-processing operations prior to registration. These are (1) correction of the uneven spacing of the A-lines caused by the nonlinear scan pattern along the fast B-scan direction; (2) correction of tilt introduced by imperfect eye alignment; (3) reduction of noise to avoid spurious correlation peaks; (4) conversion of data to single precision to fit into GPU memory and minimize processing time; (5) cropping each volume; and (6) choosing a reference volume. We summarize these pre-processing steps below.

1. Resample A-lines along the fast B-scan direction: due to nonlinearity of the scan pattern, the spacing of A-lines along the fast (X) axis direction is uneven. To correct, we resampled A-lines using a scan pattern measured on a model eye, a procedure that is sometimes referred to as “de-sinusoiding,” though we do not use a sinusoidal waveform.
2. Correct retinal tilt along the fast B-scan direction: varying displacement of the imaging beam axis relative to the center of the ocular pupil causes the images of retinal layers to exhibit differences in tilt between volumes (see Sec. 2); this variation affects both POC and NCC calculations. This tilt is numerically corrected to sub-pixel accuracy using linear interpolation.
3. Remove noise floor and convert data type: to reduce noise, volumes are normalized, scaled and converted to single precision using the following equation:
  $$V^{\prime }=\{\begin{array}{ll} 1e5 & \left(M\leq V\right)\\ \frac{V-N}{M-N}\times 1e5 & \left(N\leq V<M\right)\\ 0 & \left(V<N\right) \end{array},$$ (1)
  where V and $V^{\prime }$ are pixel values before and after normalization, N is the noise offset estimated from regions containing only non-reflective retinal structures (e.g., the vitreous), M is a constant determined by dynamic range and is typically set 35 to 40 dB above the noise offset to capture the strongest retinal reflections, and the scaling factor 1e5 is determined to avoid floating-point underflow and overflow and is a safeguard for squaring numbers and other multiplications in subsequent processing steps. We compute the noise offset N using the 95th percentile of pixel values in the vitreous for each volume.
4. Crop volume: The POC dictates that B-scans in each volume must have the same size. To maximize processing speed, we crop all volumes to the smallest size possible for a specific imaging protocol, which must be no larger than the maximum B-scan size (512×512  pixels) that can be processed by our GPGPU implementation.
5. Select reference volume: The reference volume is chosen to be the volume with the highest quality from a series of the same retinal area. Criteria for quality are minimal eye motion artifacts, high image clarity, and high signal-to-noise ratio (S/N). We developed a simple quantitative metric that encapsulates these criteria based on cross-correlation of the en face projections of adjacent fast B-scans. For each pair of adjacent lines, we store the peak correlation value r and the offset Δx at which the peak occurs. A large eye motion results in a local shearing of the image, leading to systematic variation in Δx and possibly a decrease in r; the presence of noise in the image will decrease r and increase the variability in Δx. We, therefore, define an image metric $m=\overset{´}{r}/\sigma_{r}\sigma_{\Delta x}$, where $\overset{´}{r}$ is the mean of the correlation peak heights, $\sigma_{r}$ is the standard deviation of peak heights, and $\sigma_{\Delta x}$ is the standard deviation of lateral shifts. The volume with the largest m is selected as the reference.

#### 3.1.2 Coarse registration using 3D phase correlation

**Fig. 4:**
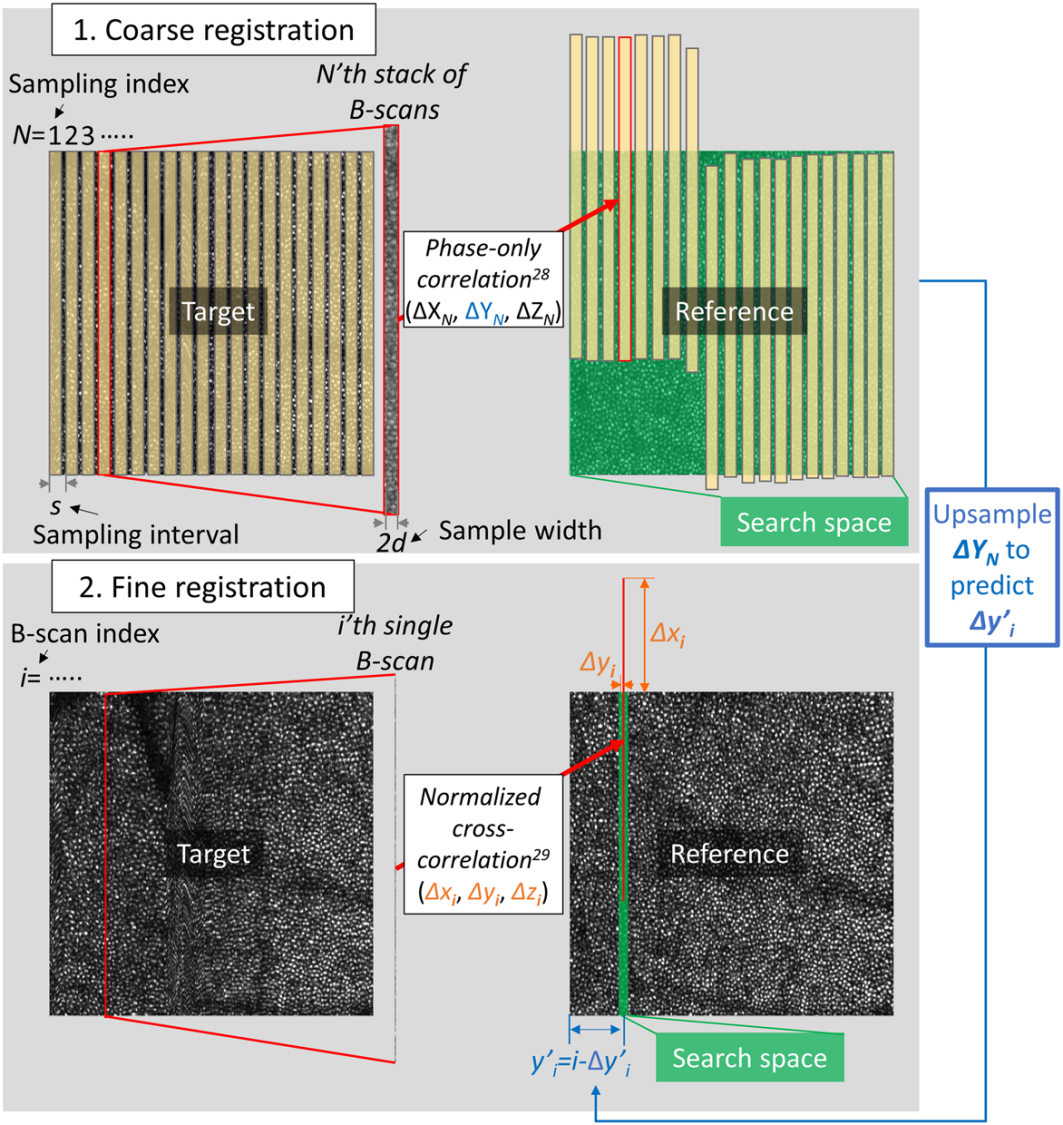
Illustration of coarse-to-fine registration. (top) Coarse registration treats each target sub-volume (stack of 16 contiguous fast B-scans) as a rigid body and finds its estimated displacement $\left(\Delta X_{N},\Delta Y_{N},\Delta Z_{N}\right)$ relative to the best-matching reference sub-volume, where N denotes the sample index (N=1,2,…). Yellow colored areas indicate 20 sampled sub-volumes. Green colored area indicates the search space. (right) To define the search space for the subsequence step of fine registration, we upsample $\Delta Y_{n}$ using a linear interpolator to predict the displacement of each target B-scan $\Delta {y^{\prime }}_{i}$, where i denotes the B-scan index. The pre-registration position of i’th B-scan is just i, so the predicted position is described as ${y^{\prime }}_{i}=i-\Delta y_{i}^{\prime }$. We then set the search space for a given B-scan as $\left({y^{\prime }}_{i}\pm w_{y}\right)$, where $w_{y}$ is a search space size, as indicated by green colored area in the bottom reference image. (bottom) Fine registration finds the accurate estimate of each fast B-scan’s location (Δx,Δy,Δz) within the narrower search space obtained from the preceding step.

We register multiple target volumes to a single reference volume using a coarse-to-fine procedure as shown in Fig. 4. For simplicity, we describe the algorithm to register a single target volume to a single reference volume. The coarse stage applies a quick 3D POC to a set of target sub-volumes (stack of contiguous fast B-scans) and finds the best-matching sub-volume in the reference volume for each target sub-volume (see Secs. 3.1.2.1 and 3.1.2.2).

##### Coarse registration sampling

To coarsely estimate the non-rigid displacements between two volumes (mainly due to eye drift), we select several sub-volumes or samples in each target volume that are evenly spaced by s and have a width of 2d along the y axis [see Fig. 4 (top left)]. Selection of optimal values for these parameters requires managing a number of tradeoffs. s must be small enough to accurately represent temporal variation in motion, but decreasing it also increases processing time. Since it is implicitly assumed that no motion occurs within a sample, increasing d runs a greater risk of violating this assumption; on the other hand, decreasing d lowers the S/N of the estimated displacement.

Our B-scan acquisition time typically ranges from 0.3 to 1 ms. We set the sampling interval s to between 5 and 30 fast B-scans, so our sample sub-volumes are temporally spaced <30  ms apart. This captures the high temporal frequency components of eye motions that are as short as 10 to 60 ms, which we have found through trial and error to be adequate for normal eye movements. Similarly, we set the sample width 2d between 8 and 16 fast B-scans, so each sample sub-volume takes 2.4 to 15 ms. This sample width is sufficiently short to freeze most eye motion^5^ yet sufficiently long for robust POC. In the presence of large, rapid eye movements, on the other hand, we can increase the robustness of POC by decreasing the sampling interval s and creating a partial overlap between sample sub-volumes (at the cost of increased processing time).

##### 3D phase-only correlation

The POC algorithm is commonly used for image registration because it is simple and is known to be well-suited for GPU implementation.^29^ We use the POC algorithm to determine the displacement of each target sub-volume relative to the best-matching reference sub-volume based on the location of the maximum value of the 3D POC [see Fig. 4 (top)].

Prior to the POC calculation, the reference volume is first zero-padded along the z and x axes to the nearest greater power of two and along the y axis to the nearest greater multiple of the sample width 2d because: (1) the POC considers an image to wrap around at the edges, so zero padding or a window function should be applied to eliminate boundary effects; (2) the FFT is faster if the size is a power of two. Then, each target sub-volume ($a_{N}$, where N is the sample sub-volume index) is zero-padded to match the size of the entire zero-padded reference volume (b), because the POC requires that both input volumes be the same size.

We compute the 3D Fourier transform of each padded target ($a_{N}$) and reference (b) volumes as shown in Eq. (2), denoted by $F(a_{N})$ and F(b), respectively. We calculate the cross-power spectrum by element-wise multiplication of $F(a_{N})$ with the complex conjugate of F(b) and normalize the result (the normalization and division are also element-wise):

$$R_{N}=\frac{F(a_{N})\circ F{\left(b\right)}^{*}}{\left|F(a_{N})\circ F{\left(b\right)}^{*}\right|},$$ (2)

We compute the inverse Fourier transform $F^{-1}(R_{N})$ to obtain a 3D cross-correlation map for each target sub-volume. The location of the maximum value in this map identifies the best-matching identically sized sub-volume within the reference volume and yields the 3D displacement of the target sub-volume $\left(\Delta X_{N},\Delta Y_{N},\Delta Z_{N}\right)$ relative to that reference sub-volume.

#### 3.1.3 Coarse prediction of individual target B-scan displacements

We use the estimated displacement $\left(\Delta X_{N},\Delta Y_{N},\Delta Z_{N}\right)$ of each target sub-volume relative to the matching reference sub-volume found in the preceding coarse registration step to narrow the search space for the following NCC-based fine registration step, as shown in Fig. 4 (bottom). Reduction of the NCC algorithm search space confers two benefits: (1) it decreases the total computation time; and (2) it increases registration accuracy by decreasing the probability of finding an incorrect match that has significant structural similarity to the target image.

To reduce the search space along the y axis, we first exclude target sub-volumes for which the displacement $\Delta Y_{N}$ could not be estimated or deviated significantly from the overall trend ($|\Delta Y_{N}-\Delta Y_{N-1}|>s$ or $|\Delta Y_{N+1}-\Delta Y_{N}|>s$). We then up-sample $\Delta Y_{N}$ using a linear interpolator to predict the displacement of individual target B-scans $\Delta {y^{\prime }}_{i}$, where i denotes the index of a B-scan within the entire volume. Displacement of target B-scans at the edges of the volume is linearly extrapolated from the nearest pair of $\Delta Y_{N}s$. The pre-registration position of i’th B-scan is just i, so its predicted position is now described as ${y^{\prime }}_{i}=i-\Delta {y^{\prime }}_{i}$. The y axis search space for a given B-scan is defined as $\left({y^{\prime }}_{i}\pm w_{y}\right)$, where the search space half-width $w_{y}$ is typically set to 8: we empirically find that the coarse shift estimate is usually within ±3 B-scans of the final estimate, so setting $w_{y}$ to eight ensures that the search space contains the matching reference B-scan.

It would be possible to also shrink the search space along the x and z axes for a given coarsely registered target B-scan based on the region of overlap between it and its best-matching reference B-scan. However, by the nature of the FFT-based NCC computation shrinking the search size requires a concomitant decrease in the size of the input data; put simply, the NCC simultaneously computes the correlation for every possible offset along x and z axes allowed by the size of the input. Shrinking the search space thus has two drawbacks. First, the overlapping region depends on the estimated displacement for each B-scan, so each FFT-based NCC computation would require different amount of memory; frequent memory re-allocation slows processing. By reducing the search space only along the y axis, we keep all B-scans the same size as the original, allowing memory to be allocated only once. Second, shrinking the search space along x and z axes would require discarding information. We choose to allow the NCC algorithm to use the full original amount of B-scan information to correct for any prediction failures from the prior coarse registration step.

#### 3.1.4 Fine registration with normalized cross-correlation

We next register individual fast B-scans of a target volume to the best-matching B-scans in a reference volume with pixel-level (not subpixel) precision using NCC^30^ [see Fig. 4 (bottom)]. Each target B-scan is cross-correlated with all reference B-scans within the reduced search space $\left({y^{\prime }}_{i}\pm w_{y}\right)$ obtained from the preceding step. That target B-scan’s final displacement estimate $\left(\Delta x_{i},\Delta y_{i},\Delta z_{i}\right)$ is the location of the maximum NCC coefficient. It is worth noting that the y component of displacement is simply the difference between B-scan indices in the target (i) and reference (j) volumes: $\Delta y_{i}=i-j$.

#### 3.1.5 Registered volume construction

After iterating the coarse-to-fine procedure described above to register other target volumes to the same reference volume, we estimate the individual fast B-scan displacement for each target volume:

$$(\Delta x_{i,T},\Delta y_{i,T},\Delta z_{i,T}),$$ (3)

where T is the target volume index. We then construct registered volumes by displacing each fast B-scan by this calculated displacement. During this process, we detect and exclude B-scans that have statistically outlying displacements or low correlation coefficients. Spurious peaks in the NCC for a single B-scan give rise to abrupt change in displacement compared to its neighbors. We detect and exclude these outliers by computing the local standard deviation of displacements $\sigma_{\Delta }$ across all B-scans within a ∼20-ms window centered on the B-scan in question and that B-scan is excluded if $\sigma_{\Delta }$ exceeds 3 to 5 pixels or its NCC correlation coefficient is lower than 0.3. These values are determined empirically so as to exclude only outliers under normal conditions and can be easily adjusted in the case of particularly severe eye movements or other abnormal conditions in which there is a breaking point (threshold) where many B-scans are thrown out.

It is worth noting that non-linear distortions caused by variation in eye velocity can result in multiple target B-scans being displaced to the same location, whereas other locations might receive no target B-scans. If multiple B-scans are displaced to the same location, the last acquired B-scan is used. If a location receives no target B-scans, a gap may appear in the registered volume [see Fig. 3 (right column)]. These gaps vary randomly between registered volumes and can be eliminated by averaging volumes as previously demonstrated;^13^ in this study, we typically average more than 100 registered volumes for each location.

### 3.2 Experimental evaluation

#### 3.2.1 Experimental design

We evaluated the performance of our 3D registration using the Indiana AO-OCT system (described by Liu et al.^31^ and Kocaoglu et al.^32^). The system ran in two-camera mode to achieve an acquisition speed of 500 K A-lines/s. The light power incident on the subject’s cornea was at or below 430  μW ($\lambda_{\text{center}}=790\;\mathrm{nm}$; $\Delta \lambda_{\mathrm{FWHM}}=42\;\mathrm{nm}$) and within American National Standards Institute-defined safe limits for our experimental protocol. The same light was used for both AO-OCT imaging and wavefront sensing. We stabilized the subject’s eye and head position to the AO-OCT system using a dental impression mounted to a motorized XYZ translation stage. All procedures adhered to the tenets of the Declaration of Helsinki and were approved by the Indiana University Institutional Review Board. Written informed consent was obtained from all subjects prior to the experiment.

As stated in the Introduction, we have successfully used our method to register AO-OCT images acquired from more than 40 subjects. Here, we illustrate the method’s performance on one of our most challenging AO-OCT datasets with images acquired at many time points over a 24-h period on a normal subject with typical fixational ability. This extreme session length introduced a number of challenges to image registration: it required many look-ins by the subject, resulting in increased eye misalignment; it tired the subject, reducing his ability to fixate over the course of the session; it necessitated repeated use of Tropicamide that varied the subject’s refractive state and tear-film state between look-ins; and it included diurnal variation in the state of the eye. These complications collectively generated a wider range of registration errors than that encountered in a typical 1 to 2-h imaging session.

We imaged a 1.5° by 1.5° patch of retina 3° temporal to the fovea in a 29-year-old subject (left eye) who was free of ocular disease and had typical fixational ability. The experiment was designed to observe cone photoreceptor disc shedding,^23^ which is a rare event (1/cone/day); we acquired three to four videos every 30 min throughout the evening of one day from 7:30 to 10:00 PM and throughout the following day from 6:00 AM to 6:00 PM. Each 5-s video contained 12 volumes acquired at 2.4  vol/s and spaced 0.41 s apart. B-scans were acquired at 1.1 KHz.

We initially registered 300 AO-OCT volumes to a reference volume containing minimal motion artifacts to evaluate registration performance. This set of volumes (34 videos acquired between 6 AM and 10:30 AM) represents a small subset of the total dataset; these are all the videos that could be registered with the 3D B-scan registration algorithm. As we will see in Sec. 4.2, registration to a global coordinate system will permit us to register the entire dataset (a total of 930 AO-OCT volumes in 110 videos.).

#### 3.2.2 Imaging quality metrics

We quantified the effectiveness of image registration using three metrics: relative power spectral contrast,^33^ image sharpness ratio (ISR),^34^ and mean squared error (MSE)^35^ computed from en face projection images of cone photoreceptors.

Relative power spectral contrast is defined as the ratio of the power spectra of two images (in this case, an average of multiple targets en face projection versus a single reference en face projection) sampled at the cone spacing period. Larger relative power spectral contrast indicates better registration because correction of eye motion must, from a practical standpoint, increase the cone mosaic regularity, thereby creating more power at the fundamental frequency of the cone mosaic.

ISR is the reduction in image sharpness in the averaged target en face projection image $I_{\mathrm{avg}}$ relative to the reference en face projection image $I_{\mathrm{ref}}$; it is defined as

$$ISR=\overset{N}{\underset{p=1}{\sum }}\|\nabla I_{\mathrm{avg}}\|/\overset{N}{\underset{p=1}{\sum }}\|\nabla I_{\mathrm{ref}}\|,$$ (4)

where ∇ denotes the spatial gradient operator (partial derivatives with respect to x and y), ‖·‖ is magnitude, and $\overset{N}{\underset{p=1}{\sum }}\ldots$ denotes summation over pixels common to both images; values range from 0 to 1, with larger values indicating better registration.

MSE is the mean squared difference in pixel value (intensity) between averaged and reference en face projection images, defined as

$$\mathrm{MSE}=\sqrt{\frac{1}{N}\overset{N}{\underset{p=1}{\sum }}{\left(I_{\mathrm{avg}}-I_{\mathrm{ref}}\right)}^{2}}.$$ (5)

Smaller MSE values indicate better registration.

We varied the number of averaged target images and examined the effect of that number on each of these three metrics. We compared: (1) the average registered-target en face projection images versus a single reference en face projection image that we judged to be of high image quality; (2) the average unregistered-target en face projection images versus the same reference image; and (3) simulated en face images composed of random pixel-correlated white noise (uniformly distributed both spatially and temporally) versus the reference. A similar comparison has been reported by Ferguson et al.^15^

#### 3.2.3 Results

**Fig. 5:**
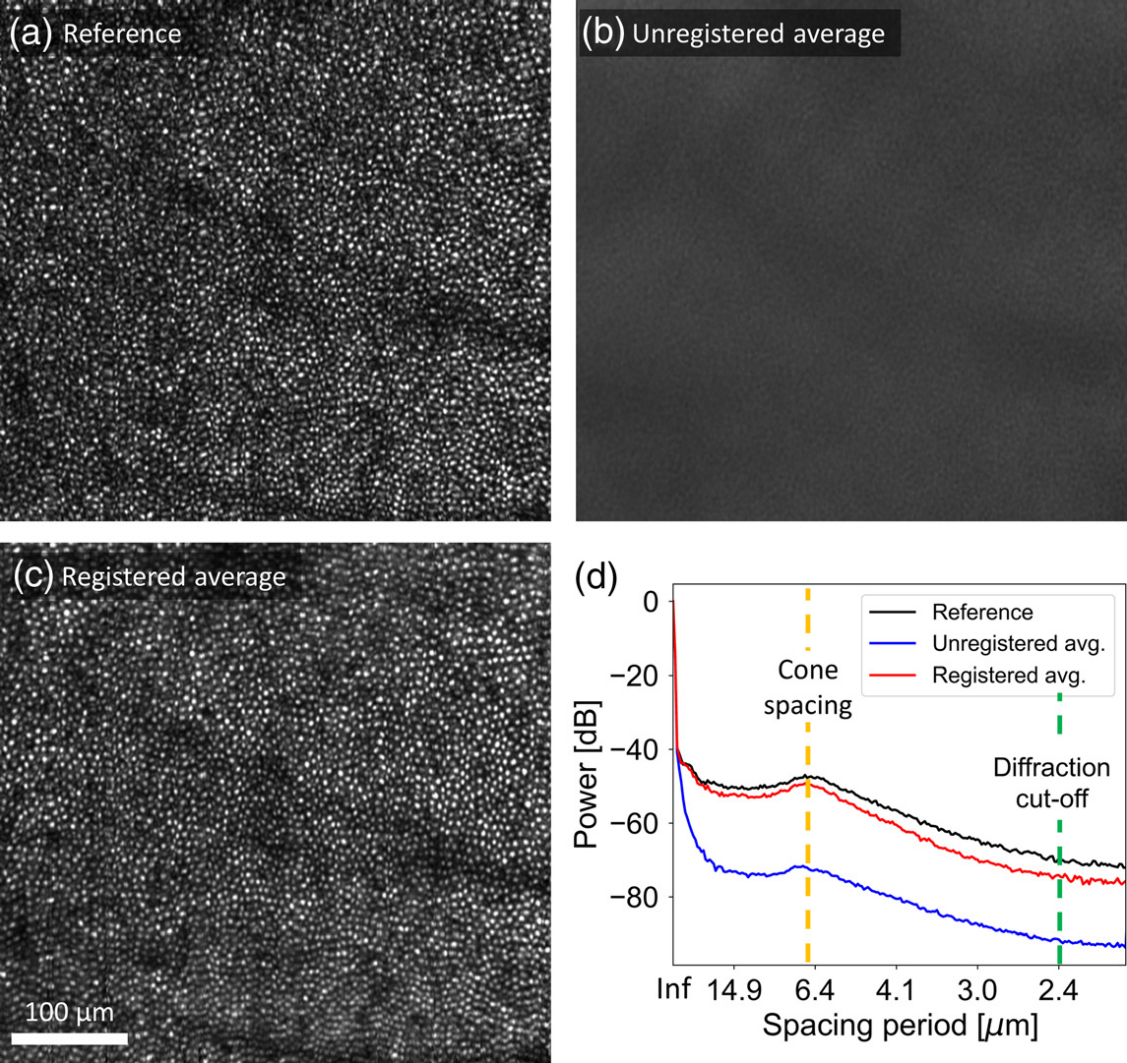
Comparison of en face projection images: (a) reference volume; (b) average of 300 unregistered target volumes; and (c) average of 300 registered target volumes. (d) Circumferential average of two-dimensional power spectrum for the reference image (black curve), average of 300 unregistered target images (blue curve), and average of 300 registered target images (red curve). Fast B-scans are oriented vertically in the images.

Figure 5 shows en face projection images of: Fig. 5(a) the selected reference volume; Fig. 5(b) average of 300 unregistered target volumes; and Fig. 5(c) average of 300 registered target volumes. The substantial difference in visibility of the cone mosaic between Figs. 5(b) and 5(c) show the power of our registration algorithm. This point is demonstrated quantitatively by plotting the circumferential average of the two-dimensional power spectrum of each image in Figure 5(d); power at the cone mosaic fundamental frequency is ∼100× greater after registration and approaches that of the single reference image. The residual degradation relative to the reference is likely caused by residual (sub-pixel) displacement errors; these cannot be removed by our registration algorithm as it registers images to the nearest whole pixel.

**Fig. 6:**
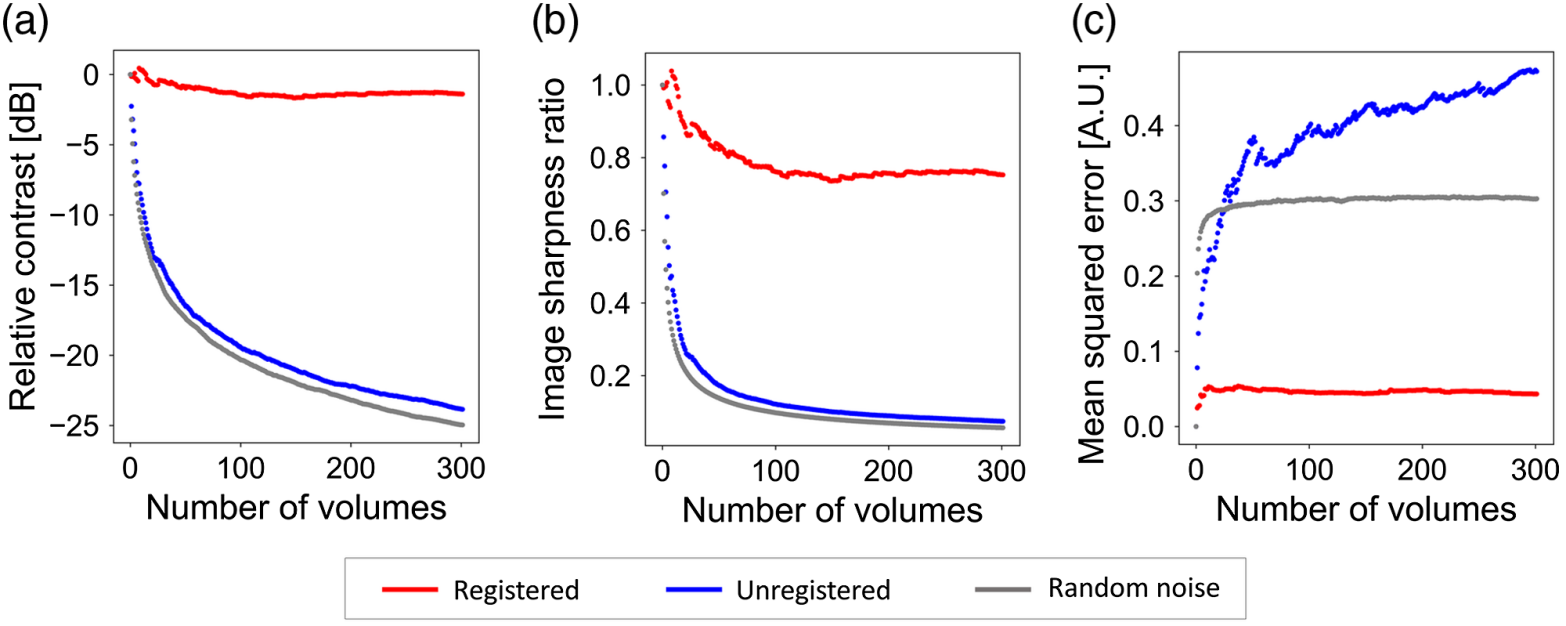
Effectiveness of the 3D B-scan registration algorithm for the same dataset used in Fig. 4. Image quality metrics, (a) relative power spectral contrast; (b) ISR; and (c) MSE, plotted as a function of number of volumes averaged.

Figure 6 shows our image quality metrics (relative power spectral contrast, ISR, and MSE) applied to the same dataset, each plotted as a function of the number of volumes averaged. As a worst-case baseline, we also plot the same metrics for simulated en face images composed of random pixel-correlated white noise (gray circles). Registered en face images have much higher relative contrast and ISR values, and much lower MSE values (better image quality) than unregistered images. Collectively, our results using relative contrast, ISR, and MSE are consistent with our visual observations and show that our 3D B-scan registration algorithm substantially enhances en face image quality by correcting motion artifacts and permitting averaging of multiple volumes.

We also evaluated registration speed for one target volume from the same dataset. Our algorithm runs much more quickly when implemented in CUDA on a GPU than in a high-level language on a CPU as we have done previously: an equivalent MATLAB implementation on a CPU (Intel Xeon CPU E5-1620 v3) registered a 450×450×300-pixel volume in 900 s, whereas our CUDA implementation on an NVIDIA GeForce GTX1080 took only 6.1 s, a 150× speed increase. Our previous 2D strip-wise registration algorithm (a combination of retinal layer segmentation and strip-wise registration) implemented in MATLAB on a CPU also took several minutes to register the same volume. The lack of a low-level language CPU-based implementation of course precludes a legitimate comparison between CPU and GPU processing. Nevertheless, we expect these speed differences to be primarily attributed to differences in the processing units.

## 4 Global A-Line Registration

Section 4.1 presents our global A-line registration algorithm for correcting torsional eye movements and image scaling and generating global motion-free coordinates. Section 4.2 describes the experimental procedure used to evaluate the performance of our approach followed by the experimental results. Sections 4.3 and 4.4 provide two applications of the algorithm to extend field of view and depth of focus, respectively.

### 4.1 Algorithm

**Fig. 7:**
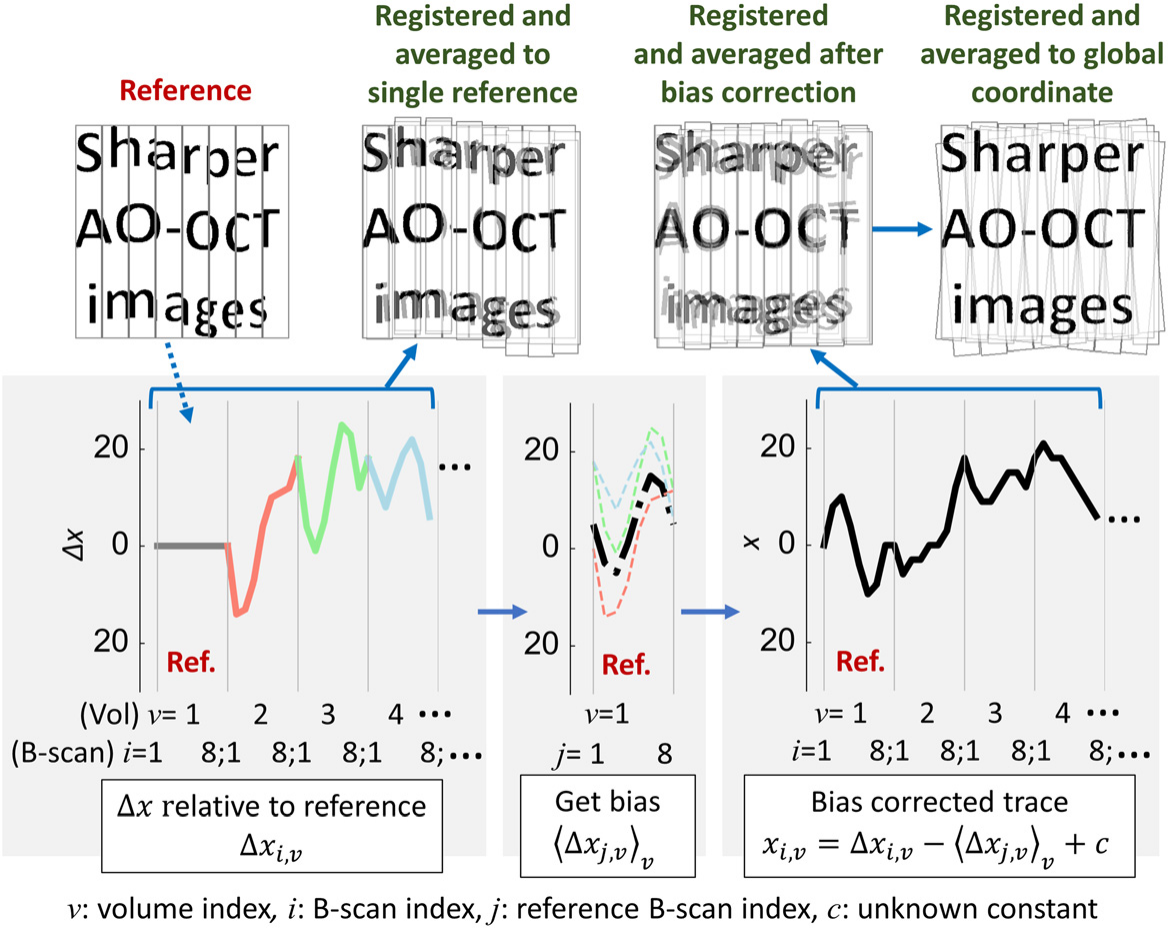
Illustration of global A-line registration. (top) For simplicity, artificial images only contain letters and are split into eight coarse strips; the actual method splits volumes into individual B-scans. Each artificial image imitates (top left) a single reference volume, (top, second column) a target volume registered to a single reference and averaged (our 3D B-scan registration algorithm; see Sec. 3.1), (top, third column) a registered and averaged image after estimating global coordinates of individual B-scans (see Sec. 4.1.2), and (top right) a registered and averaged image after estimating global coordinates of individual A-lines using affine transformation correction that removes scaling and torsional errors (see Sec. 4.1.3). (bottom) illustrates the global registration procedure to estimate global coordinates of individual B-scans for a given single reference, described in Sec. 4.1.2. For simplicity, one-dimensional motion traces are plotted in x for each image; however, the actual traces are 3D. (bottom left) B-scan shifts $\Delta x_{i,v}$ relative to the best-matching B-scan image in the reference, (bottom middle) averaging the displacements between a reference B-scan and the matched B-scans in multiple target volumes ${\overset{\to }{a}}_{j}={\left\langle \Delta x_{j,v}\right\rangle }_{v}$ for all j, (bottom right) estimating global coordinate of individual B-scans: $x_{i,v}=\Delta x_{i,v}-{\overset{\to }{a}}_{j}+c$, where c is an unknown constant.

The 3D B-scan registration algorithm discussed in Sec. 3 is effective but incomplete. As Fig. 7 shows, registered volumes still contain eye motion and torsion artifacts. First, while the algorithm removes the effects of differences in motion between target and reference volumes, it does not consider the effects of eye motion artifacts that occur during the acquisition of the reference volume itself—no reference volume is entirely free of eye motion artifacts. Second, changes in torsional eye position and shifts in head position can lead to differences in orientation and scaling between volumes that our 3D B-scan registration algorithm does not attempt to correct. Finally, some volumes are not registrable because they are temporally and spatially decorrelated by intracellular motion-induced speckle decorrelation,^20^^,^^33^ by changes in focal depth, and by orientation and scaling differences caused by eye and head motion.

We address these issues by extending a global registration method that was originally developed for 2D strip-wise registration^8^^–^^10^^,^^14^ using additional information provided in each A-scan. In Sec. 3, registration (using our 3D B-scan registration algorithm) consisted of mapping target volumes into a coordinate system defined by a reference volume. As the name implies, global A-line registration involves mapping volumes into a global coordinate system with additional constraints (described below). The approach consists of three steps [Fig. 1(b)]:

1. Separately register all target volumes to each of multiple reference volumes (Sec. 4.1.1). This yields multiple coordinate mappings for each target volume—more specifically, for each fast B-scan of each target volume—one for each reference.
2. Derive a single mapping of individual B-scans in all volumes to the multiple-reference global coordinate system (Sec. 4.1.2).
3. Correct en face affine transformations among volumes to estimate 3D global coordinates for individual A-lines (Sec. 4.1.3).

#### 4.1.1 Multiple-reference registration

Some volumes are not registrable to a single reference volume as they are temporally and spatially decorrelated, as for example due to changes in the cells’ reflectance over time and volumes acquired of non-overlapping patches of retina, respectively. We can solve this problem by selecting a new reference from the successfully registered volumes and registering all volumes (registered and non-registered) to this new reference using our 3D B-scan registration algorithm. Some or all of the volumes that could not be registered to the old reference can now be registered to the new reference; since the new reference has been registered to the original reference, any volumes registered to one are now registered to both. Thus, the number of non-registrable volumes decreases as we increase the number of reference volumes.

This multiple-reference procedure involving repeated use of our 3D B-scan registration algorithm is straightforward; the only difficulty involves the choice of reference volumes. It would be theoretically possible to use every single acquired volume in turn as a reference for all the rest, but this is rendered impractical (despite the speed of our GPU implementation) by the monotonic increase in computation time with the number of references. We instead select several reference volumes using the criteria described in Sec. 3.1.1 (step 5), with the additional requirement of a reference volume from each video. Volumes are more highly temporally correlated within videos, so choosing one reference volume from the same video as each target ensures at least one optimal-quality registration for that target. The number of reference volumes required depends strongly on the temporal and spatial correlation between volumes, so for each dataset we typically begin with five references and gradually increase the number until at least 80% of volumes can be registered.

#### 4.1.2 Global registration of individual B-scans

As mentioned previously, target volumes registered to a single reference still contain the distortions present in that reference. Stevenson and Roorda^8^ and others^9^^,^^10^^,^^14^ demonstrated that such distortions can be removed by constructing a synthetic global reference from the complete set of motion traces for each strip within a set of registered images, based on the assumption that the actual average displacement due to the eye motion is close to zero for a fixating eye (by definition, the goal of fixation is after all to maintain constant position). This permits registration of all images to a global coordinate system. We extend this global registration method to estimate the 3D global coordinates of individual B-scans using their estimated displacements relative to each reference from the preceding multiple-reference registration step.

Our registration method introduces two constraints: (1) In our scanning experimental protocol, the scanning beam moves at constant speed along the y axis. Our 3D B-scan registration algorithm implicitly incorporates the constraint that the fast B-scans are evenly spaced along the y axis, because target volumes are registered to a reference that is assumed to have this property. Since we are about to register all volumes to an empty space with a synthetic global coordinate system, we must now incorporate this constant-spacing constraint explicitly. (2) Within this global coordinate system, the ensemble of eye motions is required to have zero mean offset from the origin in all 3D. Namely, global coordinates of fast B-scans are referenced to the ensemble average of eye motions. Thus, the addition of this constraint yields maximum overlap of the B-scans at the approximate center of the averaged registered volume.

We represent the global coordinates of an individual B-scan in a target volume as ${\overset{\to }{\tau }}_{i;T}=(x_{i;T},y_{i;T},z_{i;T})$, where i is the target B-scan index, and T is the target volume index. Global coordinates of individual B-scans in a reference volume can similarly be described as ${\overset{\to }{\gamma }}_{j;R}=(x_{j;R},y_{j;R},z_{j;R})$, with j and R the reference B-scan and volume indices, respectively. The 3D B-scan registration algorithm described in Sec. 3.1 estimates the target B-scan displacements relative to the best-matching B-scans in a given reference volume $\Delta {\overset{\to }{d}}_{i,T;R}=(\Delta x_{i,T;R},\Delta y_{i,T;R},\Delta z_{i,T;R})$ [this is the same as Eq. (3) with an additional subscript added to each term to indicate the dependence on the reference volume]. We require that this relationship between target and reference B-scan positions also holds in the global coordinate system, namely:

$${\overset{\to }{\tau }}_{i,T}-{\overset{\to }{\gamma }}_{j;R}=\Delta {\overset{\to }{d}}_{i,T;R}.$$ (6)

To estimate the motion of each reference B-scan, we begin by averaging the displacements between it and the matched B-scans in multiple target volumes:

$${\overset{\to }{a}}_{j;R}={\left\langle \Delta {\overset{\to }{d}}_{j,T;R}\right\rangle }_{T},$$ (7)

where ${\left\langle \;\right\rangle }_{T}$ denotes averaging across target volumes (typically>50, which has the effect of decreasing uncertainty $\left(\sqrt{50}=\right)$ 7.1-fold relative to that of mean across a single target volume, assuming noise is uncorrelated.) and $\Delta {\overset{\to }{d}}_{i,T;R}$ is replaced with $\Delta {\overset{\to }{d}}_{j,T;R}$ using the relationship, mentioned in Sec. 3.1.4, that $j=i-\Delta y_{i,T;R}$. We can split the averaged displacements ${\vec{a}}_{j;R}$ into two terms, a zero-mean term ${\vec{b}}_{j;R}$ (i.e. ${\left\langle {\overset{\to }{b}}_{j;R}\right\rangle }_{j}=0$) and a constant residual ${\overset{\to }{c}}_{R}$:

$${\overset{\to }{a}}_{j;R}={\overset{\to }{b}}_{j;R}+{\overset{\to }{c}}_{R}.$$ (8)

The first term ${\vec{b}}_{j;R}$ remains dependent on the reference B-scan index j due to the motions within the reference volume. The second term ${\vec{c}}_{R}$ depends on the selection of reference volume for two reasons: (1) a different subset of target B-scans is registered to each reference, and (2) eye motion is a non-stationary process, i.e., its mean and variance change over time. We will revisit this issue later.

We can derive a relationship between ${\vec{b}}_{j;R}$ and the global coordinates for individual reference B-scans ${\vec{\gamma }}_{j;R}$. First, we must deal with the issue of linear scanner motion. Equation (6) demonstrates the point made earlier that we must explicitly model the y axis motion of the scanner; that motion is present in both target and reference volumes and thus vanishes from the difference. We, therefore, introduce a variable ${\overset{\to }{s}}_{i,T}=(0,i-\langle i\rangle ,0)$ representing scanner positions evenly spaced along the y axis and centered on the origin. To describe the relationship between ${\overset{\to }{b}}_{j;R}$ [which as demonstrated by Eqs. (6) and (8) cannot contain the scanner motion] and ${\overset{\to }{\gamma }}_{j;R}$ (which must contain the scanner motion), we first subtract ${\vec{s}}_{i,T}$ from ${\vec{\gamma }}_{j;R}$; we again use the relationship between i and j from Sec. 3.1.4. Second, because ${\vec{b}}_{j;R}$ by definition has zero mean, we subtract the mean of this result. Third, ${\overset{\to }{b}}_{j;R}$ has the opposite sign to ${\vec{\gamma }}_{j;R}$, [this follows from its definition and Eq. (6)], so we negate it, yielding:

$${\overset{\to }{b}}_{j;R}=-[{\overset{\to }{\gamma }}_{j;R}-{\overset{\to }{s}}_{i,T}-{\left\langle {\overset{\to }{\gamma }}_{j;R}-{\overset{\to }{s}}_{i,T}\right\rangle }_{j}].$$ (9)

In other words, ${\overset{\to }{b}}_{j;R}$ yields the global reference B-scan position ${\vec{\gamma }}_{j;R}$ minus the (known) scanner motion ${\vec{s}}_{i,T}$ and an unknown constant ${\left\langle {\overset{\to }{\gamma }}_{j;R}-{\overset{\to }{s}}_{i,T}\right\rangle }_{j}$.

The global target coordinates ${\vec{\tau }}_{i,T}$ are related to the global reference coordinates ${\vec{\gamma }}_{j;R}$ by Eq. (6). To solve for ${\vec{\tau }}_{i,T}$ we introduce:

$${\overset{\to }{p}}_{i,T;R}=\Delta {\overset{\to }{d}}_{i,T;R}-{\overset{\to }{a}}_{j;R}+{\overset{\to }{s}}_{i,T}.$$ (10)

Substituting Eqs. (6)–(9) into Eq. (10) and solving for ${\vec{\tau }}_{i,T}$ yields:

$${\overset{\to }{\tau }}_{i,T}={\overset{\to }{p}}_{i,T;R}+{\left\langle {\overset{\to }{\gamma }}_{j;R}-{\overset{\to }{s}}_{i,T}\right\rangle }_{j}+{\overset{\to }{c}}_{R}.$$ (11)

We have two constant terms, one known (${\vec{c}}_{R}$) and the other unknown (${\left\langle {\overset{\to }{\gamma }}_{j;R}-{\overset{\to }{s}}_{i,T}\right\rangle }_{j}$). We combine them into a single unknown ${\overset{\to }{c^{\prime }}}_{R}={\overset{\to }{c}}_{R}+{\left\langle {\overset{\to }{\gamma }}_{j;R}-{\overset{\to }{s}}_{i,T}\right\rangle }_{j}$, yielding:

$${\overset{\to }{\tau }}_{i,T}={\overset{\to }{p}}_{i,T;R}+{\overset{\to }{c^{\prime }}}_{R}.$$ (12)

This provides the target B-scan global coordinates ${\vec{\tau }}_{i,T}$. It is worth noting that this also provides the reference B-scan global coordinates ${\overset{\to }{\gamma }}_{j;R}$, by simply replacing (i,T) with (j,R), and so we use ${\vec{\tau }}_{i,T}$ to represent both target and reference B-scan global coordinates below.

However, both terms on the right-hand side in Eq. (12) depend on the reference volume R; essentially we have as many unknowns ${\overset{\to }{c^{\prime }}}_{R}$ as we have equations, preventing a straightforward closed-form solution. Instead, we derive an iterative numerical solution as follows: first, we split the unknown constant ${\overset{\to }{c^{\prime }}}_{R}$ into two terms, one of which (${\overset{\to }{\varepsilon }}_{R}$) depends on R and the other ($\overset{\to }{\epsilon }$) does not. Then, Eq. (12) can be rewritten as

$${\overset{\to }{\tau }}_{i,T}={\overset{\to }{p}}_{i,T;R}+{\overset{\to }{\varepsilon }}_{R}+\overset{\to }{\epsilon }.$$ (13)

We impose an initial condition on ${\vec{\varepsilon }}_{R}$: ${\vec{\varepsilon }}_{R_{0}}=(0,0,0)$, i.e., we select a reference volume $R_{0}=1$ for which we force ${\vec{\varepsilon }}_{R}=(0,0,0)$. This constraint can be equivalently stated as ${\overset{\to }{\varepsilon }}_{R}={\overset{\to }{p}}_{i,T;R_{0}}-{\overset{\to }{p}}_{i,T;R}$, or more usefully as ${\overset{\to }{p}}_{i,T;R}+{\overset{\to }{\varepsilon }}_{R}={\overset{\to }{p}}_{i,T;R_{0}}$, i.e., it is constant with respect to R. We use this property to implement an iterative algorithm that minimizes differences in ${\overset{\to }{p}}_{i,T;R}+{\overset{\to }{\varepsilon }}_{R}$ across reference volumes R. First, we find a set of B-scans $i\in W_{R,R^{\prime }}$ that are registered to two arbitrary reference volumes R and $R^{\prime }(R\neq R^{\prime })$. Second, we replace ${\vec{\varepsilon }}_{R}$ with a minimization variable ${\overset{\to }{∍}}_{R}$ and compute the sum of squared differences between ${\overset{\to }{p}}_{i,T;R}+{\overset{\to }{∍}}_{R}$ and ${\overset{\to }{p}}_{i,T;R^{\prime }}+{\overset{\to }{∍}}_{R^{\prime }}$, first across B-scans within the set $i\in W_{R,R^{\prime }}$ and then across all possible combinations of references R and $R^{\prime }$.

The magnitude of this sum of sums is the cost function $f(R,R^{\prime })$:

$$f(R,R^{\prime })=\|\overset{N}{\underset{R^{\prime }=1}{\sum }}\overset{N}{\underset{R=1}{\sum }}\underset{i\in W_{R,R^{\prime }}}{\sum }{\left({\overset{\to }{p}}_{i,T,R}-{\overset{\to }{p}}_{i,T,R^{\prime }}+{\overset{\to }{∍}}_{R}\;-{\overset{\to }{∍}}_{R^{\prime }}\right)}^{2}\|.$$ (14)

Our iterative minimization algorithm finds ε→R^=argmin∍R→f(R,R′) that minimizes the cost function $f(R,R^{\prime })$ and yields a unique solution. However, we are left with a residual error e→i,T,R=ε→R^−ε→R that we minimize by averaging across reference volumes. Substituting our estimators into Eq. (13), we have

τ→i,T=⟨p→i,T,R+ε→R^−e→i,T,R⟩R+ϵ→=o→i,T+ϵ→, (15)

where o→i,T=⟨p→i,T,R+  ε→R^−e→i,T,R⟩R. At this point, we apply our constraint that the B-scan global coordinates should have zero mean (i.e., ${\left\langle {\overset{\to }{\tau }}_{i,T}\right\rangle }_{i,T}=0$) and replace $\overset{\to }{\epsilon }$ by $-{\left\langle {\overset{\to }{o}}_{i,T}\right\rangle }_{i,T}$, yielding

$${\overset{\to }{\tau }}_{i,T}=\;{\overset{\to }{o}}_{i,T}-{\left\langle {\overset{\to }{o}}_{i,T}\right\rangle }_{i,T}.$$ (16)

This equation expresses in deceptively simple form the dependence of B-scan global target coordinates on the estimated target-reference displacements, subjected several times to a zero-mean constraint and explicitly incorporating scanner motion. In Sec. 4.1.3, we extend this estimate to global coordinates of individual A-lines and in Sec. 5.1 use this theory to compare our method to others.

#### 4.1.3 Global registration of individual A-lines

Compared to our 3D B-scan registration algorithm outlined in Sec. 3.1, the procedure just described permits registration of more volumes and minimizes local translation errors of individual B-scans. However, the resulting registered and averaged volumes are still distorted due to eye movements around, e.g., the line of sight (torsion), and in depth (which cause scaling errors). Figure 7 (top right) shows the effect of these errors. To correct them, we first make the assumption that we can ignore torsion and scaling errors between volumes within a single video (∼5  s).^10^^,^^28^ We generate registered en face projection images based on the estimated global coordinates for individual B-scans using Eq. (16) and average subsets of these images taken from the same video and selected to fill in any gaps between B-scans. These averaged image subsets are used to estimate affine transformations between videos. We estimate and remove these errors by iteratively optimizing image similarity using Mattes’ metric,^36^ specifically using the MATLAB (The MathWorks, Natick, 2017) function *imregtform*, which estimates the geometric transformation that best aligns two 2-D or 3-D images. The image subset containing the volume with the lowest variance in ${\vec{\tau }}_{i,T}$ [the B-scan global coordinates, Eq. (16)] is selected as the reference for this stage. Each of the other en face image subsets is passed to *imregtform* along with this reference image subset; the function yields a 3×3 homogeneous-coordinates (x,y,w)^37^^,^^38^ affine transformation matrix $A_{T}$ for each volume, representing translation, rotation, and scaling in the xy plane relative to the reference. This approach is reliable when image features are high-contrast and temporally stable, as in the case of the cone mosaic; however, it tends to fail if these conditions do not hold, e.g., while imaging other retinal layers or a damaged retina. For these cases, we estimate the affine transformation by iterating over different image subsets and applying other image similarity metrics such as the masked cross-correlation coefficient;^30^ this is an ongoing research topic. After we estimate the transformation matrix $A_{T}$ for each volume, we augment each $A_{T}$ with a fourth row and column to represent transformations in the third spatial dimension (z; depth) using homogeneous 3-D coordinates (x,y,z,w).^37^^,^^38^ Multiplying each of these matrices by its corresponding target volume’s B-scan coordinates applies a different displacement to each A-line, permitting us to assign a global coordinate vector to each individual A-line in every acquired volume.

We represent the global coordinates of an individual A-line in a volume as ${\overset{\to }{\chi }}_{\alpha ,i,T}=(x_{\alpha ,i;T},y_{\alpha ,i;T},z_{\alpha ,i;T})$, where α is the new A-line index and i and T are the B-scan and target volume indices as before. We can now derive ${\vec{\chi }}_{a,i,v}$ from ${\vec{\tau }}_{i,T}$ [the B-scan global coordinates, Eq. (16)]. We introduce a new variable ${\overset{\to }{\varsigma }}_{\alpha }=(\alpha -\langle \alpha \rangle ,0,0)$ to represent linear scanner motion along the fast-scan x axis (as we did previously for the slow-scan y axis, see Sec. 4.1.2). ${\vec{\varsigma }}_{\alpha }$ represents scanner positions evenly spaced along the x axis and centered on the plane x=0; we add it to ${\vec{\tau }}_{i,T}$ and convert the sum to homogeneous coordinates as mentioned above. Finally, we multiply each such vector by the appropriate affine transformation matrix $A_{T}$, yielding the global coordinates of individual A-lines in homogenous coordinates:

$${\overset{\to }{\chi }}_{\alpha ,i,T}=A_{T}\cdot ({\overset{\to }{\tau }}_{i,T}+{\overset{\to }{\varsigma }}_{\alpha }),$$ (17)

where · denotes matrix multiplication.

### 4.2 Experimental Evaluation

#### 4.2.1 Experimental design

We used the dataset from Sec. 3.2 to demonstrate and evaluate the performance of the global A-line registration algorithm. As we mentioned in that section, we could only register a subset of videos using the 3D B-scan registration algorithm. With the addition of global registration, we are able to register all 110 videos collected in this experiment, for a total of 930 volumes. The duration of the experiment, and hence the maximum time elapsed between videos, was 22 h and 30 min. We selected 25 reference volumes and used them to register all volumes to global coordinates with the global A-line registration algorithm (described in Sec. 4.1).

#### 4.2.2 Results

**Fig. 8:**
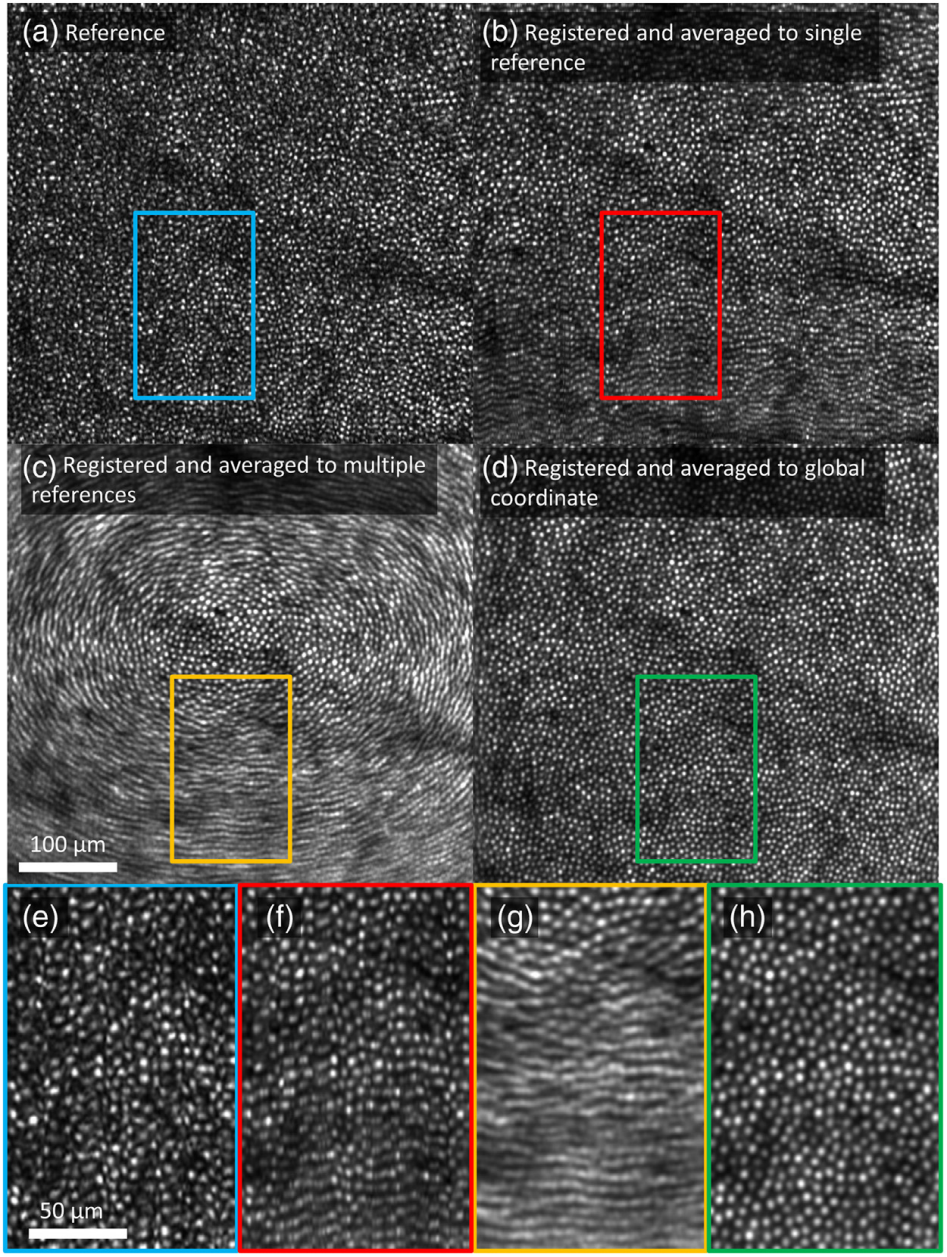
Comparison of en face projection images of: (a) a reference volume; (b) average of 676 volumes registered to a single reference; (c) average of 930 volumes registered to 25 references; and (d) average of 880 volumes registered to 25 references and corrected for torsion and scaling. (e)–(h) Magnified view (2×) of corresponding en face projection images. Fast B-scans are oriented vertically in the images. The 1.5° by 1.5° patch of retina is located 3° temporal to the fovea. Video 1 (MPEG, 22 MB [URL: https://doi.org/10.1117/1.JBO.26.1.016001.1]) highlights the effect of averaging on en face projection images that are registered to (b) a single reference; (c) 25 references; and (d) a global coordinate, by increasing the number of averaged frames.

Figure 8 shows en face projection images at various stages in the registration process. Portions of the image enclosed in colored rectangles are displayed with magnification at the bottom of Fig. 8 to better display the differences among them.

Figure 8(a) shows a single reference image. Even though we selected a reference volume with minimal eye motion, distortions presumably due to tremor motion are visible as irregularity of the cone mosaic and of the shapes of the individual cones.

Figure 8(b) shows the average of the all of the targets after registration to a single reference using the 3D B-scan registration algorithm (described in Sec. 3.1). The cone mosaic appears slightly less irregular than in Fig. 8(a), but uncorrected torsional eye movements cause a blurry strip along the bottom edge of the image. More generally, the top and bottom edges are prone to torsional blur at this stage whereas the left and right edges are not due to the vertical orientation of the fast B-scans [see an example in Fig. 7 (top, second column)]. At left and right sides, torsion creates roughly uniform motion parallel to the fast B-scans that are removed by the 3D B-scan registration algorithm. At top and bottom, on the other hand, torsion creates motion perpendicular to the B-scans and in opposite directions that cannot be removed by the algorithm. In the image shown in Fig. 8, the reference’s torsional axis is displaced toward the top of the image, which causes the bottom edge to be more visibly blurred. It is worth noting that the blur is more significant in Fig. 8(b) than in Fig. 5(c) as we registered more videos (676 volumes compared to 300 volumes), many of which could not be accurately registered with the 3D B-scan registration algorithm because of increased torsion present.

Figure 8(c) shows what happens when we average after registering all 930 target volumes to global coordinates using 25 reference volumes but without correcting for the effects of torsional and scaling eye movements. This uses the portion of the global A-line registration algorithm described in Secs. 4.1.1 and 4.1.2 [see an example in Fig. 7 (top, third column)]. Registering to multiple reference volumes that differ in torsion relative to one another cancels out the false vertical displacements introduced at left and right edges by the 3D B-scan registration algorithm in response to torsion, creating radially symmetric torsional blur.

Figure 8(d) shows the effect of applying the affine transformation described in Sec. 3.1.3, the final stage of the global A-line registration algorithm [see an example in Fig. 7 (top, fourth column)]. This yields a more regular, uniform cone mosaic made up of rounder cones. At this point, each A-line of every volume (corresponding to pixels in these projected images) has been individually transformed into global coordinates.

Table 1 summarizes our image quality metrics (defined in Sec. 3.2.2) applied to each averaged image. The relative power spectral contrast—the ratio of power evaluated at the cone spacing period—improved by 1.37 dB (37% increase) by the global A-line registration algorithm. This suggests that the cone mosaic in the image is more regularly spaced after global registration than in any single reference image. The global A-line registration algorithm also improved the ISR value—the reduction in image sharpness due to averaging—by 0.64 dB (16% increase). However, neither metric captures the notable improvement in the cone mosaic regularity (uniform spacing) and clarity of individual cones (roundness of spots) as evident by visual inspection of the images in Figs. 8(b) and 8(d). These differences (in sampling regularity and cone clarity) are better captured by S/N [ratio of spectral power at the cone spacing period to the noise floor (high-frequency tail of the spectrum)^33^] that shows a 9.4 dB (771%) improvement between the two images (rightmost column of Table 2). Comparison of the signal and noise computed separately along the fast and slow B-scan axes reveal similar power at the cone spacing period using both 3D B-scan registration and global A-line registration (always around −24.5+/−0.5  dB). Comparison of the noise level between registration methods and scan axes is far more interesting, as it carries information about sampling regularity. We assume that sampling is already regular during fast B-scans because they are too short to be distorted by eye and head movement, so there should be no effect of registration algorithm on the noise floor in the fast B-scan direction. By contrast, if our global A-line registration reduces sampling irregularity of the cone mosaic, it should decrease the noise floor along the slow B-scan axis relative to 3D B-scan registration. Both predictions are correct: we find a noise floor of ∼−52  dB for both 3D B-scan and global A-line registration in the fast B-scan direction; along the slow B-scan axis, on the other hand, we have noise levels of −39.4 and −48.9  dB for 3D B-scan and global A-line registration, respectively. This indicates that global A-line registration significantly improves sampling regularity along the slow B-scan axis.

**Table 1:** Image quality metrics for the global A-line registration algorithm.

	Relative power spectral contrast (dB)	ISR	SNR (dB)
Registered and averaged to single reference	−1.044	0.781	27.98
Registered and averaged to global coordinate	0.330	0.906	37.38
Improvement	1.3 (37%)	0.64 dB (16%)	9.4 (771%)

**Fig. 9:**
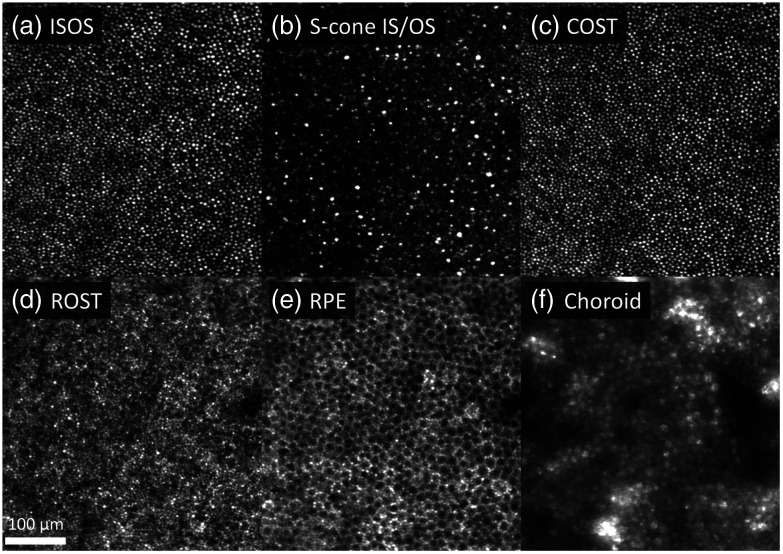
En face projection images of retinal layers at (a) IS/OS of M and L cone photoreceptors; (b) IS/OS of S cone photoreceptors; (c) COST; (d) ROST; (e) RPE; and (f) choroid layers. It is worth noting that S cones possess a longer IS than M and L cones^39^ resulting in a slightly deeper IS/OS reflection.^40^ Furu Zhang confirmed the deeper S-cone reflection using his cone classification dataset in Zhang et al.^21^ (personal communication, April 2019). All volumes were registered with the global A-line registration algorithm to the same global coordinates and averaged. The 1.5° by 1.5° patch of retina is located 3° temporal to the fovea, which is to the left of the image. Superior retina is up. Fast B-scans are oriented horizontally in the images. Video 2 (MPEG, 16 MB [URL: https://doi.org/10.1117/1.JBO.26.1.016001.2]) is a fly-through movie of the outer retina that compares three different reconstructions from the same dataset: reference, registered, and averaged to a single reference, and registered and averaged to global coordinate.

Using the global A-line registration algorithm, we were able to resolve detailed structure of the outer retina with minimum distortion as shown in Fig. 9. En face projection images were computed for each of the following cellular structures: the medium- and long-wavelength (M and L) cone inner segment/outer segment junction (IS/OS), short-wavelength (S) cone IS/OS, cone outer segment tips (COST), rod outer segment tips (ROST), and retinal pigment epithelium (RPE). The associated video (Video 2) highlights that fine details are more evident and regularly spaced compared to the single-reference approach (3D B-scan registration algorithm), leading to more accurate morphometric analysis. In addition, the power of multiple references to increase volume averaging is clearer in the ROST and RPE. Speckle noise was significantly reduced, and the images have better contrast despite the weaker reflections of these tissues.

The global A-line registration algorithm also improved visualization of the underlying choroid but in an unexpected way as shown in Fig. 9(f). The apparent mosaic pattern in the choroid is actually a shadow pattern created by the AO-OCT beam penetrating more readily through the darkened centers as opposed to brighter surrounds of the overlying mosaic of RPE cells. This surreal polka-dot-like pattern matches exactly the RPE cell pattern [compare Figs. 9(e) and 9(f)] and is more evident when multiple references are used to increase volume averaging (Video 2).

### 4.3 Application 1: Extending Field of View

#### 4.3.1 Experimental design

Without the global A-line registration algorithm, field of view is limited to the area of overlap of the target volumes with the single reference. We can use the fact that the global coordinate system of the global A-line registration algorithm combines registration results for several reference volumes to extend the field of view beyond the original volume size by selecting reference volumes that are displaced from one another. We used a dataset acquired at 5° temporal to the fovea (right eye of the same subject used in Sec. 3.2) during the experiment described in Sec. 3.2 to demonstrate and evaluate this capability. We selected 30 reference volumes that were displaced from one another and using the metric described in Sec. 3.1.1 (step 5), combined all registration results using the method described in Sec. 4.1, and finally generated a single average registered volume from 630 target volumes taken from 81 videos.

#### 4.3.2 Results

**Fig. 10:**
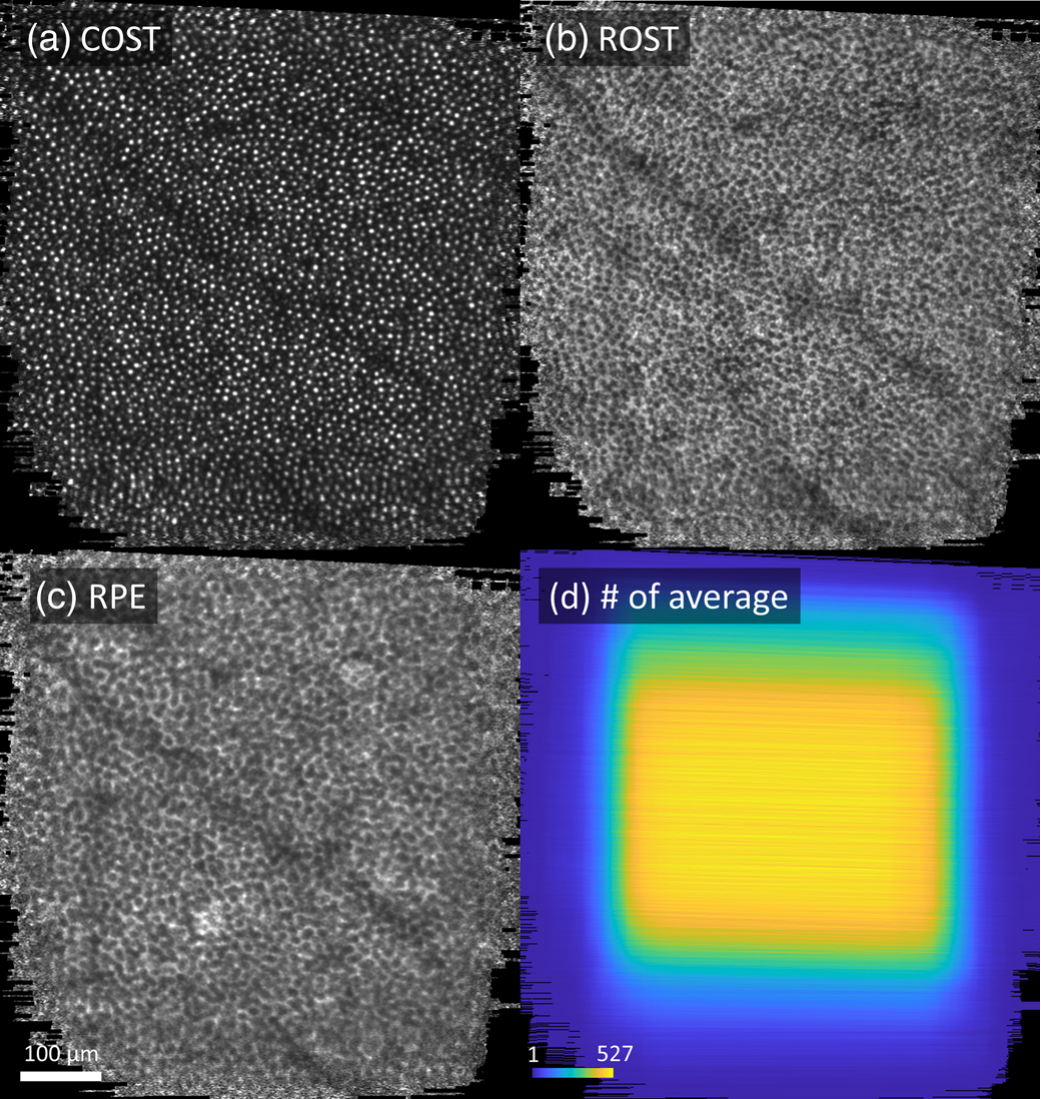
Extended field of view en face images at (a) COST; (b) ROST; and (c) RPE layer, which were extracted from the single average volume. Color image (d) depicts the spatial distribution of the number of overlapped pixels present in multiple registered volumes. Images were acquired at 5° temporal to the fovea.

Figure 10 shows extended field of view en face images extracted from the single average registered volume, showing the (a) COST, (b) ROST, and (c) RPE layers. The effective field of view ($0.41\;{\mathrm{mm}}^{2}$) was 2.1× larger than that of the original volumes ($0.19\;{\mathrm{mm}}^{2}$). Despite subject fixation there was significant translation between videos and smaller but still significant drift within videos. The number of pixels found in multiple registered images (i.e., overlapped pixels) was greatest at the center near fixation and decreased toward the edge, as shown in Fig. 10(d) leading to greater eye-movement estimation accuracy and image quality around the center [Figs. 10(a)–10(c)]. However, cone photoreceptor and RPE cell mosaics are clearly visible across the extended field of view [Figs. 10(a)–10(c)].

### 4.4 Application 2: Extending Depth of Focus

#### 4.4.1 Experimental design

The shallow depth of focus of AO-OCT (∼50  μm) has limited our ability to simultaneously image retinal layers at different depths with high lateral resolution; we can collect separate volume images of the same retinal region that are focused on different retinal layers, but we could not previously register them to one another. For example, it was previously extremely difficult to register a volume focused on the photoreceptors to one focused on the ganglion cells. Optical solutions such as axicon lens or addition of high-order spherical aberrations have been proposed to extend the depth of focus,^41^^–^^43^ but these necessarily come at the expense of decreased signal strength and resolution. However, we can use the global coordinate system of the global A-line registration algorithm to align volumes focused at different depths by selecting a set of reference volumes that span the range of depths (a.k.a, focus stacking).

To demonstrate and evaluate this ability to enhance depth of focus, we acquired AO-OCT volumes at 3° temporal to the fovea in the right eye of a 26-year-old subject, who is different from the subject in Sec. 3, was free of ocular disease and had a typical fixational ability. Focus depth was varied relative to the photoreceptors from −23 to 208  μm (posterior to anterior) in 23-μm steps and 10 or more AO-OCT volume videos were acquired for each depth. We selected 25 reference volumes spanning the depth range, combined all registration results using the global A-line registration algorithm described in Sec. 4.1, and averaged 1394 registered volumes to obtain a single volume with preserved focus across that entire range. We repeated the experiment on a second subject and obtained similar results (not shown). This subject was 31 years old, free of ocular disease, and had a typical fixational ability.

#### 4.4.2 Results

**Fig. 11:**
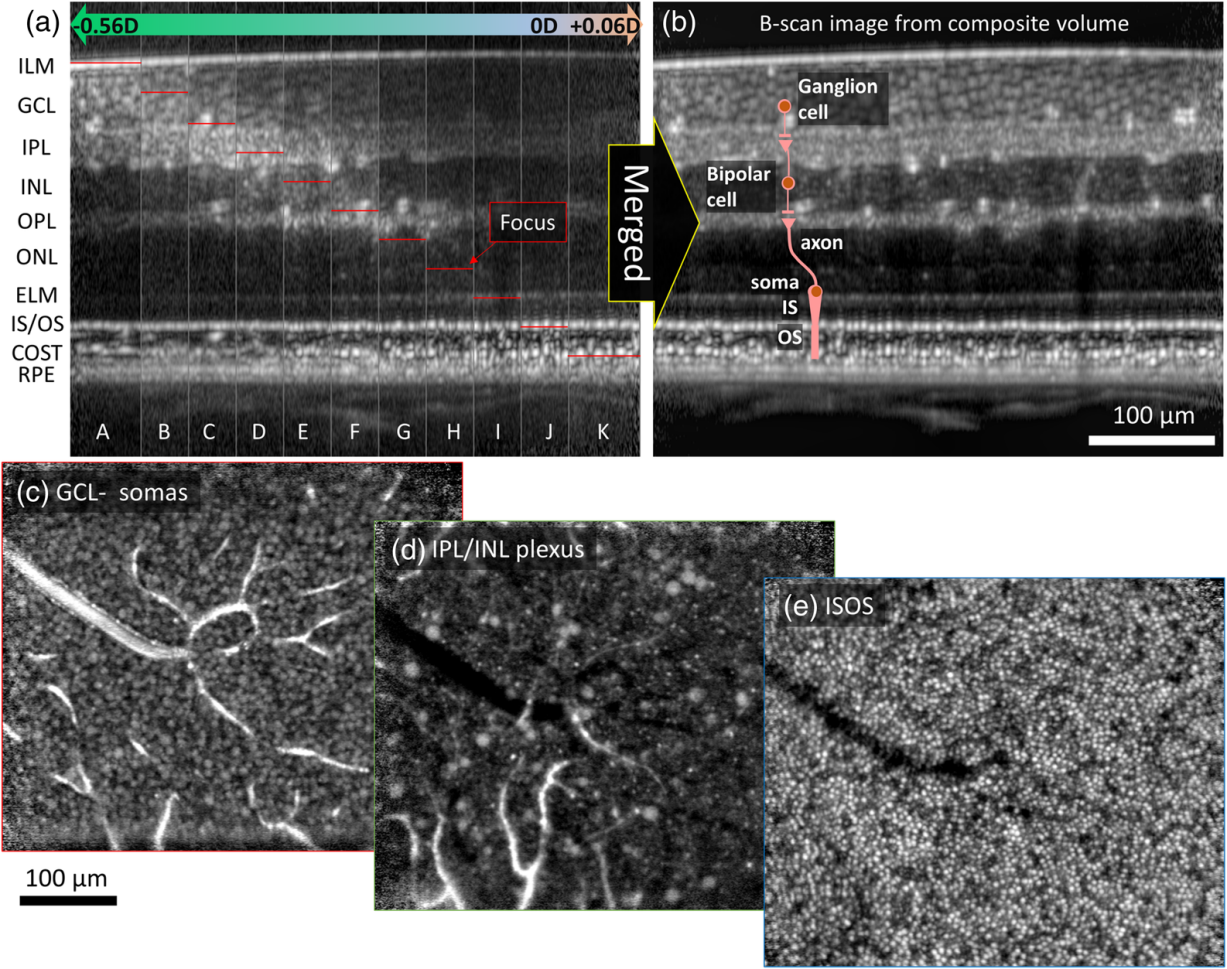
Increased depth of field without compromising resolution. Cross-sectional images show effect of focusing at different depths, obtained with the global A-line registration algorithm and multiple references focused at different depths: (a) prior to and (b) after merging by simply averaging. Thin red lines indicate the focus positions in strips A-K. The extended depth of focus traverses the entire retinal thickness. (c) En face images at GCL; (d) IPL/ INL plexus; and (e) IS/OS layer are shown, which were extracted from the single average volume in (b). Video 3 (MPEG, 13 MB [URL: https://doi.org/10.1117/1.JBO.26.1.016001.3]) is a fly-through movie of the same volume, showing globally-registered en face images at all depths. We applied mu-law compression (MATLAB™ function *compand*^44^) and a local Laplacian filter (MATLAB™ function *locallapfilt*^45^^,^^46^) to compensate for the high dynamic range of the AO-OCT images (>30  dB).

The effect of shifting the narrow focus peak in depth is clearly shown in Fig. 11(a); different retinal layers have increased visibility and sharpness in different vertical strips representing different focus depths. The benefit of using our global A-line registration algorithm to combine registered volumes focused on different retinal layers is clearly shown in Fig. 11(b); all retinal layers are in sharp focus across the composite volume. The effective depth of focus of the combined volume is estimated as ∼280  μm (= 23-μm focal shift × 10 steps + 50  μm depth of focus), which is 5.6× larger than that of each single volume (∼50-μm depth of focus).

## 5 Discussion

We present a new registration method that cascades a 3D B-scan registration algorithm with a global A-line registration for correcting motion artifacts in AO-OCT volume images. Our low-level GPU implementation of the first algorithm speeds up processing time by 150× over a CPU-based MATLAB implementation, permitting us to average hundreds of images of a single location for improved S/N without sacrificing sharpness or contrast (Sec. 3.2.3). We quantify this improvement using three image quality metrics (Secs. 3.2.2 and 3.2.3). In addition, we demonstrate that the second algorithm minimizes distortion and improves the regularity of the cone photoreceptor mosaic in the averaged image and supports enhanced visualization of low-contrast cellular retinal features by registering and averaging several hundreds of volume images down to the level of individual A-lines (Sec. 4.2.2). The use of multiple-reference global coordinates followed by an affine transformation permits (1) extending the field of view by 2.1× (Sec. 4.3.2) and (2) extending depth of focus by 5.6× (Sec. 4.4.2).

We show several key examples that illustrate the performance of our registration algorithm. We have found that it is robust enough to provide reliable registration for most subjects, including patients with diseased retinas exhibiting, e.g., ganglion cell layer thinning, drusen, edema, and photoreceptor loss and degeneration. We routinely use the method in our laboratory’s imaging studies, now applied to more than 40 subjects across many retinal eccentricities and time points (data not shown in this paper but some can be found elsewhere^4^^,^^20^^–^^25^).

### 5.1 Comparison to Other Registration Methods

Stevenson and Roorda^8^ first developed a 2D strip-wise registration algorithm to register images from AO-SLO to a synthetic global reference. As we will discuss, we have previosuly adapted this algorithm to AO-OCT volumes because their en face projections are distorted similarly to AO-SLO images. However, the slower volume image acquisition rate of AO-OCT (<10  Hz, compared to >30  Hz for AO-SLO) results in more severe distortions. AO-OCT volume images are also subject to z axis distortion due to axial eye motions that Stevenson and Roorda’s strip-wise registration does not correct. These factors make AO-OCT volume registration more challenging.

Our 3D B-scan registration algorithm uses a coarse-to-fine registration approach with a combination of phase correlation and NCC to increase the processing speed and 3D registration accuracy (see Sec. 3). Do^7^ (2016, master’s thesis, Section 9) found that our method is more robust to fast microsaccadic eye movements than previous 2D strip-wise approaches.^12^^,^^13^ Our method is also simpler than some previous approaches in that it does not require identification of specific features (e.g., blood vessels for feature-based registration^47^^–^^49^) or segmentation of particular retinal layers to estimate eye motion, making it readily applicable to raster-scanned OCT images. This consideration should also make our approach more robust to retinal deformation caused by, e.g., edemas or drusen that make both layer segmentation and feature identification more difficult.

The global portion of our registration algorithm differs from all of the earlier approaches that we will discuss below in that it combines results from multiple reference images to handle eye-motion-induced distortions present within any single reference image and scaling/torsion errors created by changes in eye and head position between target volumes (see Sec. 4). Use of multiple references permits us to extend the strip-wise estimation of 2D global coordinates demonstrated by Stevenson and Roorda^8^ and others^9^^,^^10^^,^^14^ to estimation of 3D global coordinates for each individual A-line.

Stevenson and Roorda^8^ first introduced a method to correct the motion within a single reference image based on the reasonable assumption that during fixation the average displacement is close to zero. They also corrected torsional error by dividing each horizontal image strip in half and measuring the difference in vertical displacement between the two halves. The results were validated by comparing with eye-position measurements using a dual-Purkinje tracker. Their approach was clearly successful, but they omitted most of the details of the calculation, making a direct comparison difficult. We have attempted to describe our approach as completely as possible, allowing for accurate evaluation of our assumptions and constraints and facilitating detailed comparison.

Vogel et al.^9^ utilized a map-seeking circuit algorithm to register images from AO-SLO. This is a type of template-matching algorithm that uses the principle of superposition to simultaneously and independently solve for different transformational components. Using strips from a single reference image as templates and computing independent transformations for each strip permits detection of image warping. Vogel et al. used this method to track eye motion in real time,^50^ but with a reduced search space as small as a single strip. They also followed Stevenson and Roorda’s method to correct the motion within a reference image and then estimate the target strip global coordinate. To estimate the motion of each strip, they averaged the displacements between it and strips that were measured at a constant frame rate. Recall that in Eq. (8) we compute a variable ${\vec{a}}_{j;R}$, which for a given reference B-scan is the average displacement across all target volumes relative to the best-matching B-scan in each target volume. The averaging process used by Vogel et al. is equivalent to instead computing ${\overset{\to }{a}}_{i;T}={\left\langle \Delta {\overset{\to }{d}}_{i,T}\right\rangle }_{T}$, which is the average across target volumes for B-scans at the same position in each target volume of the displacement of the best-matching reference B-scan. They then used this ${\overset{\to }{a}}_{i;T}$ instead of ${\overset{\to }{a}}_{j;R}$ in Eq. (11) to yield the target strip global coordinate (target B-scan global coordinate in our case). However, this approach is valid only if the magnitude of y axis displacement between reference and target strips is smaller than the strip width (the number of lines per strip), in which case i=j. This is because ${\overset{\to }{a}}_{j;R}$ must depend on the reference strip (or B-scan, in our case) index j, not on the target strip index i, to be related to the global coordinate for a reference strip. In our case, we estimate global coordinates for each B-scan, which corresponds to a strip that is only one pixel wide; any y axis displacement larger than a pixel disobeys the requirement that j=i. We solved this issue using the relationship $j=i-\Delta y_{i,T;R}$ (Sec. 3.1.4), permitting us to compute global coordinates for single B-scans even in the presence of large y axis displacements. We also explicitly correct rotation and scaling errors to yield global coordinates for individual A-lines.

Bedggood and Metha^10^ used a strip-wise registration algorithm to register simulated AO-SLO images from high-speed AO flood illumination (200 Hz). The simulated AO-SLO images were generated as follows: First, they computed the translational shifts between AO flood illumination images and upsampled 5× to generate quasi-eye movements (from 200 to 1000 Hz). Second, they temporally downsampled AO flood illumination images (from 200 to 20 Hz) to generate quasi AO-SLO images. Finally, they divided each quasi AO-SLO image into 50 strips and translated the strips based on the quasi-eye movement to simulate the distorted AO-SLO image. The results were validated by comparing with an actual AO flood illumination image. They estimated the motion within the single reference image using two different approaches: “simplified” and “combined.” The simplified approach is equivalent to using ${\vec{a}}_{i;T}$ instead of ${\vec{a}}_{j;R}$ to estimate the motion within the reference image (such as Vogel’s approach). The combined approach is equivalent to using ${\vec{a}}_{j;R}$ (like our approach). Performance of the combined approach was superior to that of the simplified approach. As we stated in the preceding paragraph, it is only correct to use ${\vec{a}}_{i;T}$ to estimate motion in a reference image and target image global coordinates under certain very restricted conditions. They also used multiple references but only for validation purpose and did not combine them to estimate the global coordinate. Like Vogel et al., Bedggood and Metha do not account for rotation and scaling errors, but their much shorter measurement times (∼5  s total, compared to several hours in our case) may render this correction unnecessary.^28^

Azimipour et al.^14^ used a strip-wise registration algorithm to register AO-SLO and AO-OCT images and estimate the motion within a reference image. They validated the method using a simulated cone mosaic and simulated eye movement (see Ref. 11 for details about the model used), but did not compare the outputs to a ground truth. They used the simplified approach of Bedggood and Metha (in our terms, averaging target-reference displacements use ${\vec{a}}_{i;T}$ instead of ${\vec{a}}_{j;R}$) to estimate and remove the motion within a reference image; they then repeated the basic strip-wise procedure using the single motion-corrected reference. Our algorithm does not require such a second round of registration, because we directly estimate global coordinates of multiple target images using ${\vec{a}}_{j;R}$ and the relationship $j=i-\Delta y_{i,T;R}$ (Sec. 3.1.4). Like the previous authors, Azimipour et al. did not account for rotation and scaling errors.

It is important to note that the errors due to axial and torsional eye movements are much less salient when volumes are registered to a single reference volume [see Figs. 8(b) and 8(f)]. We found, however, that the correction of these errors was critical for improving image quality and maximizing the number of registrable volumes [see Figs. 8(d) and 8(h)]. We claim that although single-reference image registration significantly improves image quality, the results are generally distorted both in terms of feature locations and shapes, potentially leading to errors in estimation of densitometry and morphology of the retina. In addition, combining registration results from multiple reference images provides the novel benefits of permitting us to significantly extend both field of view and depth of focus (Secs. 4.3 and 4.4). Finally, it is also important to note that global registration is accomplished during post-processing and thus does not require additional hardware to track eye movements^6^^,^^15^^–^^17^ or a change in scan pattern,^18^^,^^19^ either of which would add complexity and cost.

### 5.2 Limitations of Our Approach

Our approach assumes that fast B-scans are rigid because fast B-scans are captured in a short enough time (0.3 to 1 ms) to contain negligible eye- and head-motion artifacts. Based on this assumption, we correct for uneven A-scan sampling and retinal tilt along the fast B-scan direction (see Sec. 3.1.1). This removes most scanner distortions and motion artifacts in fast B-scan images. However, the assumption of constant motion for all A-lines within each B-scan is clearly violated when registering B-scans that contain large blood vessels because they are highly dynamic, with flow along the vessel at rates of up to ∼35  mm/s. Also, eye movement velocity varies continuously in all directions even during a fast B-scan image acquisition. Drift and tremor velocities are negligible (<0.3  μm/B-scan), but microsaccades (traversing several dozens and hundreds of photoreceptors over a duration of ∼25  ms) are not and so prevent accurate estimation of motion at these time points. In addition, it remains difficult to reliably register B-scans in volumes that contain multiple saccades as saccades interfere with the coarse prediction of target B-scan displacements (Sec. 3.1.3).

Our approach requires memory-intensive computing to use an entire reference volume as a search space. We carefully manage memory allocation to quickly process large datasets and so reduce the required memory down to 5 GB of GPU RAM for processing a 512×512×512  pixel volume. GPUs containing this much RAM remain expensive. In addition, the speed of our implementation is currently limited by the time required for writing pre-processed volumes to disk and not by the algorithm itself. This suggests that data compression, implemented downstream of the algorithm, would yield significant speed improvements.

As quantified in Sec. 4.2.2, the combined registration method (3D B-scan registration plus global A-line registration) minimizes distortion and improves the regularity of cone photoreceptor mosaic in the averaged image significantly more than the 3D B-scan registration algorithm alone; this improvement is particularly evident in the 9.4 dB increase in S/N. However, due to the inaccessibility of the ground truth—we have no independent source of undistorted high-resolution retinal images—we are unable to strictly assess the accuracy of the results. Instead, we thoroughly describe our algorithm, allowing the accurate evaluation of our assumptions and constraints and facilitating detailed comparison.

It is also important to note that the global A-line registration algorithm corrects both torsional eye movements and image scaling; absent of these corrections, the registered and averaged images are significantly distorted [Fig. 8(c)]. This effect is exaggerated by the long duration (a day) of the experiment described in Sec. 4.3, but the distortions are evident at the edges of registered volumes, even in a 15-min experiment that requires multiple look-ins. Therefore, this final step will likely benefit many of today’s AO-OCT experiments in terms of improved registration accuracy. It is worth noting, however, that we only correct torsion and scaling errors relative to their average values. It may under some circumstances be possible to use images from other imaging modalities as a source of information about absolute orientation and scale.^10^

## 6 Conclusion

We present a new image registration method that cascades a 3D B-scan correlation-based algorithm with a multiple-reference global A-line registration method for correcting eye movement artifacts in AO-OCT volume images; global A-line registration corrects eye motion artifacts separately for each individual A-line. Our GPU implementation is 150× faster than our previous CPU implementation. The combination of algorithms achieves high registration accuracy and robustness and enhanced image quality as measured by a variety of metrics and permits significant expansion of the field of view and depth of focus of the final registered image.
