# Multi-reference global registration of individual A-lines in adaptive optics optical coherence tomography retinal images (Publisher’s Note)

**DOI:** 10.1117/1.JBO.26.1.019803

**Published:** 2021-01-25

**Authors:** Kazuhiro Kurokawa, James A. Crowell, Nhan Do, John J. Lee, Donald T. Miller

**Affiliations:** a Indiana University, School of Optometry, Bloomington, Indiana, United States; b Indiana University–Purdue University, Purdue School of Engineering and Technology, Indianapolis, Indiana, United States; c Google, Mountain View, California, United States

## Abstract

The note corrects an error that appeared in Equation 14 of the originally published article.

This article [*J. Biomed. Opt.* **26**(1), 016001 (2021) doi: 10.1117/1.JBO.26.1.016001] was originally published on 6 January 2021 with an error in Eq. (14). Due to editorial oversight, two blank spaces appeared where two variables should have appeared.

Original error:

$$f(R,R^{\prime })=\|\overset{N}{\underset{R^{\prime }=1}{\sum }}\overset{N}{\underset{R=1}{\sum }}\underset{i\in W_{R,R^{\prime }}}{\sum }{\left({\overset{\to }{p}}_{i,T,R}-{\overset{\to }{p}}_{i,T,R^{\prime }}+{\overset{\to }{⏧}}_{R}-{\overset{\to }{⏧}}_{R^{\prime }}\right)}^{2}\|.$$ (14)

Corrected:

$$f(R,R^{\prime })=\|\overset{N}{\underset{R^{\prime }=1}{\sum }}\overset{N}{\underset{R=1}{\sum }}\underset{i\in W_{R,R^{\prime }}}{\sum }{\left({\overset{\to }{p}}_{i,T,R}-{\overset{\to }{p}}_{i,T,R^{\prime }}+{\overset{\to }{∍}}_{R}-{\overset{\to }{∍}}_{R^{\prime }}\right)}^{2}\|.$$ (14)

The error was corrected on 8 January 2021.

