# Supplementary material for: Multi-reference global registration of individual A-lines in adaptive optics optical coherence tomography retinal images
Source: J Biomed Opt. 2021 Jan 6;26(1):016001. doi: 10.1117/1.JBO.26.1.016001 (PMC7787477; doi:10.1117/1.JBO.26.1.016001)
Supplement: Supplementary file 1 [file JBO_026_016001_SD001.pdf]

Supplemental Section A

Compared one-to-one, GPU cores are less powerful than CPU cores. However, GPUs typically have thousands of cores, so highly parallelizable tasks can run much faster on a GPU. A parallelizable task requires the same computation to be performed many times with different inputs with all instances of the computation being independent of one another. Our 3D B-scan registration algorithm is parallelizable because it carries out several tasks that can be computed independently of all of the others, including zero-padding, the computation of product or sum of two matrices, and matrix transposition. In addition, the correlation-map computations for POC and NCC are based on the 3D Fast Fourier Transform (FFT) and many parallel 2D FFTs, respectively. Both computations themselves are highly parallelizable. We implemented these tasks in CUDA (Computer Unified Device Architecture; NVIDIA, Santa Clara) and its language. The details of the implementation are described by Do[7] and are beyond the scope of this manuscript. However, it is worth noting that our algorithm benefits particularly from NVIDIA GPUs because the correlation-map computations use the performant cuFFT CUDA library[51].

A brute-force implementation of our 3D registration algorithm would require more memory than is available on current GPUs. Making algorithm run quickly without exceeding available memory requires careful optimization of memory allocation and caching of intermediate results. The input volume size is known, so memory can be allocated at the beginning and reused or overwritten as required. We also carefully identify duplicate computations and cache their results for re-use. The POC uses the same reference volume for all target volumes, so computations specific to the reference can be performed once and the results re-used with multiple targets; target volumes are sequentially copied to the GPU and registered to the reference. This reduces the amount of memory required to register a set of 512 × 512 × 512-pixel volumes down to 3.1GB. The NCC would also benefit from caching of the FFT results of all fast B-scans of the reference volume; however, NCC requires more memory than POC, so this would far exceed GPU memory capacity. We therefore only cache the fast B-scan images within the moving search space determined by the first-stage POC computation. The FFT results are deleted from the cache as their corresponding B-scan images leave the current search space and are replaced by FFT results of B-scans entering the search space.

As a result, we reduce the total memory required for both of the above computations (POC and NCC) to less than 5 GB for a volume size of 512 × 512 × 512 pixels.
